# Molecular characterization of the acquisition of longevity during seed maturation in soybean

**DOI:** 10.1371/journal.pone.0180282

**Published:** 2017-07-12

**Authors:** Juliana Joice Pereira Lima, Julia Buitink, David Lalanne, Rubiana Falopa Rossi, Sandra Pelletier, Edvaldo Aparecido Amaral da Silva, Olivier Leprince

**Affiliations:** 1 Faculdade de Ciências Agronômicas, Universidade Estadual Paulista Júlio de Mesquita Filho, Botucatu, São Paulo State, Brazil; 2 Institut de Recherche en Horticulture et Semences, INRA, Agrocampus Ouest, Université d’Angers, SFR QUASAV, Beaucouzé, France; University of Manitoba, CANADA

## Abstract

Seed longevity, defined as the ability to remain alive during storage, is an important agronomic factor. Poor longevity negatively impacts seedling establishment and consequently crop yield. This is particularly problematic for soybean as seeds have a short lifespan. While the economic importance of soybean has fueled a large number of transcriptome studies during embryogenesis and seed filling, the mechanisms regulating seed longevity during late maturation remain poorly understood. Here, a detailed physiological and molecular characterization of late seed maturation was performed in soybean to obtain a comprehensive overview of the regulatory genes that are potentially involved in longevity. Longevity appeared at physiological maturity at the end of seed filling before maturation drying and progressively doubled until the seeds reached the dry state. The increase in longevity was associated with the expression of genes encoding protective chaperones such as heat shock proteins and the repression of nuclear and chloroplast genes involved in a range of chloroplast activities, including photosynthesis. An increase in the raffinose family oligosaccharides (RFO)/sucrose ratio together with changes in RFO metabolism genes was also associated with longevity. A gene co-expression network analysis revealed 27 transcription factors whose expression profiles were highly correlated with longevity. Eight of them were previously identified in the longevity network of *Medicago truncatula*, including homologues of *ERF110*, *HSF6AB*, *NFXL1* and members of the *DREB2* family. The network also contained several transcription factors associated with auxin and developmental cell fate during flowering, organ growth and differentiation. A transcriptional transition occurred concomitant with seed chlorophyll loss and detachment from the mother plant, suggesting the activation of a post-abscission program. This transition was enriched with AP2/EREBP and WRKY transcription factors and genes associated with growth, germination and post-transcriptional processes, suggesting that this program prepares the seed for the dry quiescent state and germination.

## Introduction

Soybean is one of the most important oil crop species for food, feed and a range of industrial applications. Producing highly vigorous seeds is a key lever to increase crop production. Seed longevity, defined as the ability to remain alive during storage under dry conditions, is an important agronomic factor in the preservation of seed fitness after harvest [1]. Poor longevity leads to unexpected losses in seed viability during storage and negatively impacts seedling establishment and crop yield [1, 2]. This is particularly problematic for soybean as seeds have a short lifespan during storage, especially in humid and tropical environment [2–4]. In addition, longevity is pivotal to ensure the preservation of our genetic resources through dry seeds of crops and wild species [5, 6].

Longevity is conferred by the ability to stabilize the biological entity for long periods of time by the formation of an amorphous highly viscous, solid-like matrix (i.e. a glassy state) in the cells that suspends integrated metabolic activities and severely slows down deteriorative reactions [2, 7, 8]. Seed longevity is also attributed to a range of protective compounds [9, 10], including non-reducing soluble sugars (sucrose (Suc) and raffinose (Raf) family oligosaccharides, RFO [11, 12]) and a set of late embryogenesis abundant (LEA) proteins and heat shock proteins (HSP) [13–15]. Together with sugars, both types of proteins act as chaperones and molecular shields to prevent protein denaturation and membrane destabilization during drying and in the dry state. Longevity is also conferred by antioxidant mechanisms that limit oxidation of lipids, proteins and nucleic acids during storage such as glutathione [16 and references therein], tocopherols [17], flavonoids that are present in the seed coat [18] and lipocalins [19]. Several repair mechanisms also contribute to longevity when they are activated during seed imbibition to fix damage that occurred to proteins and DNA during storage [20, 21].Next to protection and repair, an impaired degradation of chlorophyll appears to negatively affect longevity [12, 22]. The presence of chlorophyll is considered as an indicator of immaturity but how it affects longevity remains unsolved.

To be commercially successful, crop seeds should be harvested when longevity reaches its maximum [1, 10]. In legumes, longevity is progressively acquired during seed maturation from seed filling onwards [10, 23–25]. In soybean, there exist conflicting data as to whether seed longevity reaches a maximum at seed filling [4, 26] or later during maturation [25, 27]. Delaying harvest to obtain maximum longevity increases the risk of exposing mature seeds to rapid deterioration in the field due to high humidity and temperature [25–27]. The mechanisms regulating the acquisition of seed longevity and vigor during late maturation remain poorly understood. Hence, the dearth of knowledge of late seed maturation programs remains an obstacle to commercial production of high quality seeds.

In soybean, transcriptome studies generated a wealth of data describing seed development during embryogenesis and filling [28–33]. However, there is little information on transcriptome changes occurring in soybean during late seed maturation, when longevity is acquired. The purpose of this study was to provide a physiological and molecular characterization of the soybean seed maturation using RNAseq to obtain a comprehensive overview of the regulatory genes that are potentially involved in seed longevity. Our data show that a developmental program is activated during late maturation that is more complex than a simple arrest of seed filling and seed drying.

## Material and methods

### Plant material and seed physiology

**Fig 1:**
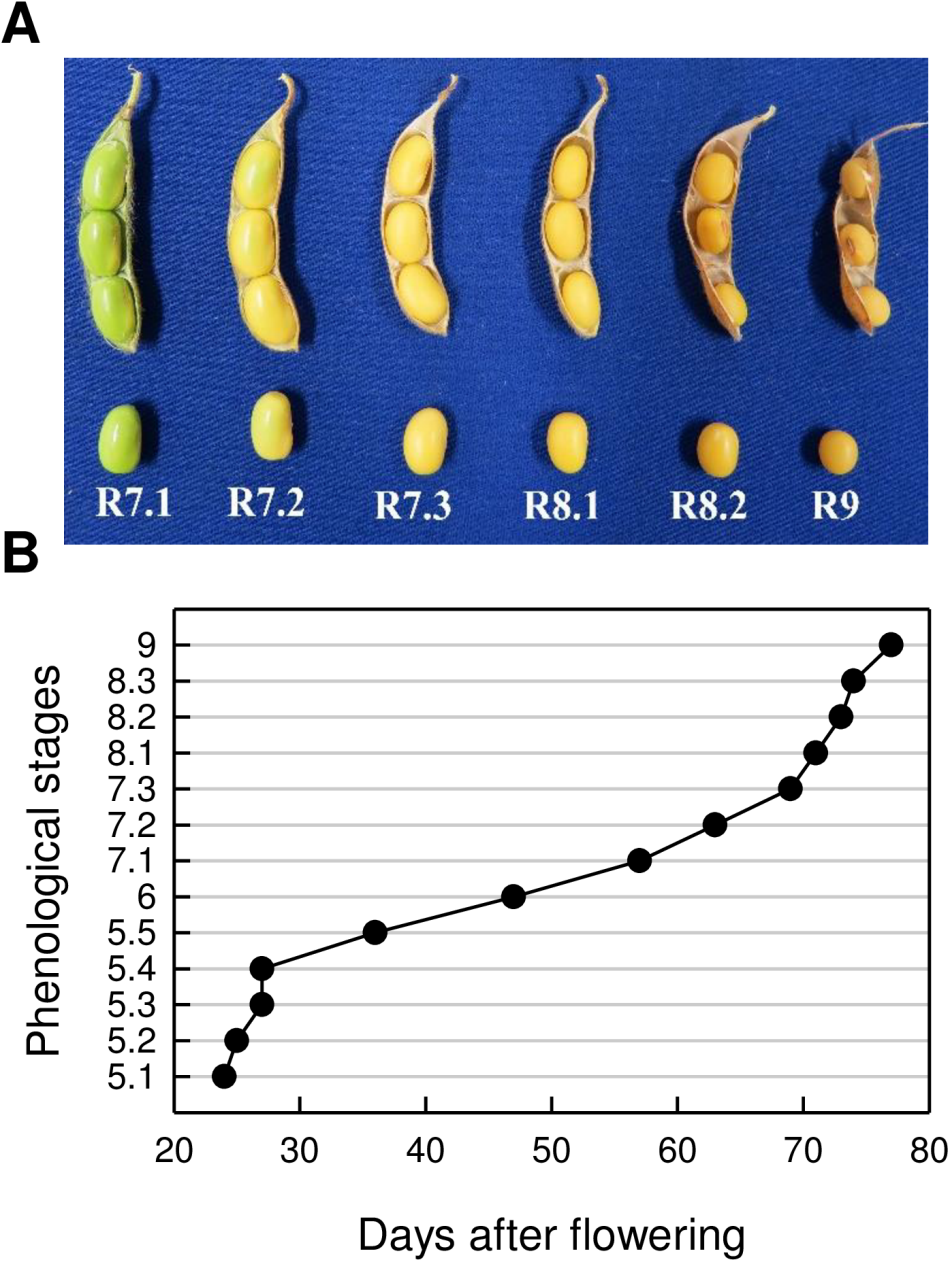
Seed and pod development of soybean. (A) Seed phenological stages during the acquisition of seed longevity (2013 crop). (B) The relationship between seed age and phenological stages (2014 crop). Stage 9 corresponds to dry mature seeds.

Soybean plants (*Glycine max* L. cv. BRS284) were grown using standard planting and cultural techniques in the experimental farm of the São Paulo State University in Botucatu (Brazil) during two consecutive years (2012/2013; 2013/2014). Seed development was monitored using phenological stages [34] and flower tagging (for 2014 only). Intermediate phenological stages between at R7 and R8 (for both years) were incorporated in order to obtain a higher precision of the time course of acquisition of physiological quality attributes after seed filling (S1 Table). The relationship between seed age and phenological stages is shown in Fig 1. Pods were manually removed and seeds were used immediately. For germination assays, 4 replicates of 25 seeds were imbibed in moistened paper rolls at 25°C. To test desiccation tolerance, seeds harvested at different stages were subjected to fast drying by incubation at 40% RH at 30°C until they reached a moisture content of 10% (dry weight basis, DW) (i.e. after 2 days). Thereafter, seed germination was assayed as described above. To assess longevity, immature artificially dried and mature seeds were stored in the dark at 35°C at 75% RH using hermetically closed containers containing a saturated NaCl solution. The water content of the seeds at these conditions was 0.13 g H_2_O/g DW^-1^. At different time intervals, 4 replicates of 25 seeds were retrieved and imbibed as described above, and final germination percentage was counted.

### Soluble sugar assay

Soluble sugar contents were assessed separately in cotyledons and embryonic axes from the phenological stage R6 onwards using DIONEX-HPLC according to Rosnoblet et al. [35]. Analysis was performed on triplicates of 6 axis and cotyledons.

### RNA sequencing, quality control and reads alignment

Developing and mature seeds harvested from up to 200 plants of the 2014 crop at each stage were frozen in liquid nitrogen. Total RNA was extracted using the NucleoSpin® RNA Plant kit (Macherey-Nagel) according to the manufacturer instructions. Total RNA from high quality samples (RIN values > 8.9 evaluated by a 2100 Bioanalyzer, Agilent Technologies, Santa Clara, CA, USA) were used for library preparation sequencing at the Laboratório Central de Tecnologias de Alto Desempenho em Ciências da Vida (LaCTAD) from the University of Campinas, Brazil. cDNA libraries were generated using the TruSeq RNA sample preparation kit (Illumina, San Diego, CA, USA). After estimation of the insert size of the libraries and quantification using quantitative PCR, samples were diluted and pooled. Three lanes were sequenced using a HiSeq2500 (Illumina) with the TruSeq SBS Kit v3-HS, according to the manufacturer instructions. Sequencing adaptors and low complexity reads were removed in an initial filtering step. After quality control, reads were mapped to the ‘Williams 82’ soybean reference genome (assembly Glyma.Wm82.a1.0, annotation v2.0) using Bowtie2 [36]. RNAseq data were deposited in the NCBI Gene Expression Omnibus database (accession no. GSE98199).

### Transcriptome and gene network analysis

Estimation of differential gene expression and statistical analyses were performed using DESeq2, v1.11.21 [37] available as a R Bioconductor package. Genes were retained as differentially expressed when the ratio was at least two-fold and the P-value adjusted for multiple testing using the Benjamini-Hochberg (BH) method <0.05. Relative expression data were normalized by dividing the mean normalized gene expression value obtained of the stage n+1 by the value obtained of stage n. Functional enrichment of gene ontology (GO) was performed using the GO enrichment tool of Soybase (https://www.soybase.org) with the Glyma 2.0 gene model.

Data used for the generation of the transcription factor network corresponded to genes encoding transcription factors (TF) that varied in their expression profiles during development. A total of 754 TF were retained with transcripts levels showing a variance >1 throughout seed development. The gene co-expression network was constructed using the Expression Correlation Plugin for Cytoscape, with a Pearson correlation coefficient (PCC) cutoff of 0.97, only including positive correlations. Gene interactions were visualized using the open source software Cytoscape (version 2.8.1) using an organic layout. Identification of genes correlated with longevity was obtained using the trait based gene significance measure [24, 38] where the gene significance of a gene equals the absolute correlation between the gene expression profile and longevity expressed as P50 (days to obtain 50% germination during storage).

### Quantitative PCR

RNA extraction was performed as previously described on three biological replicates of 30 seeds. First strand cDNA was synthesized from 2 μg total RNA using High Capacity RNA-to-cDNA kit (Applied Biosystems, place) following manufacturer’s instructions. Quantitative real time PCR was performed on a thermocycler Eco Real-Time (Illumina) with SYBR Green qPCR ReadyMix (Sigma Aldrich, place) using the manufacturer’s instructions. Data were analyzed with EcoStudy program version 5.0 (Illumina). Primer efficiency was calculated as described in Ruiter et al. [39]. Relative expression levels were calculated using the comparative 2△(Ct) method [40] using two reference genes, 20S proteasome subunit beta (Glyma.06G078500) and 60S ribosomal protein L6 (Glyma.15G271300) [41]. In our RNAseq dataset, transcript levels of both genes showed little variation (variance of 0.035 and 0.08 respectively). Forward and reverse primers used for these genes are listed in S2 Table.

## Results

### Seed longevity is acquired at the end of maturation

**Fig 2:**
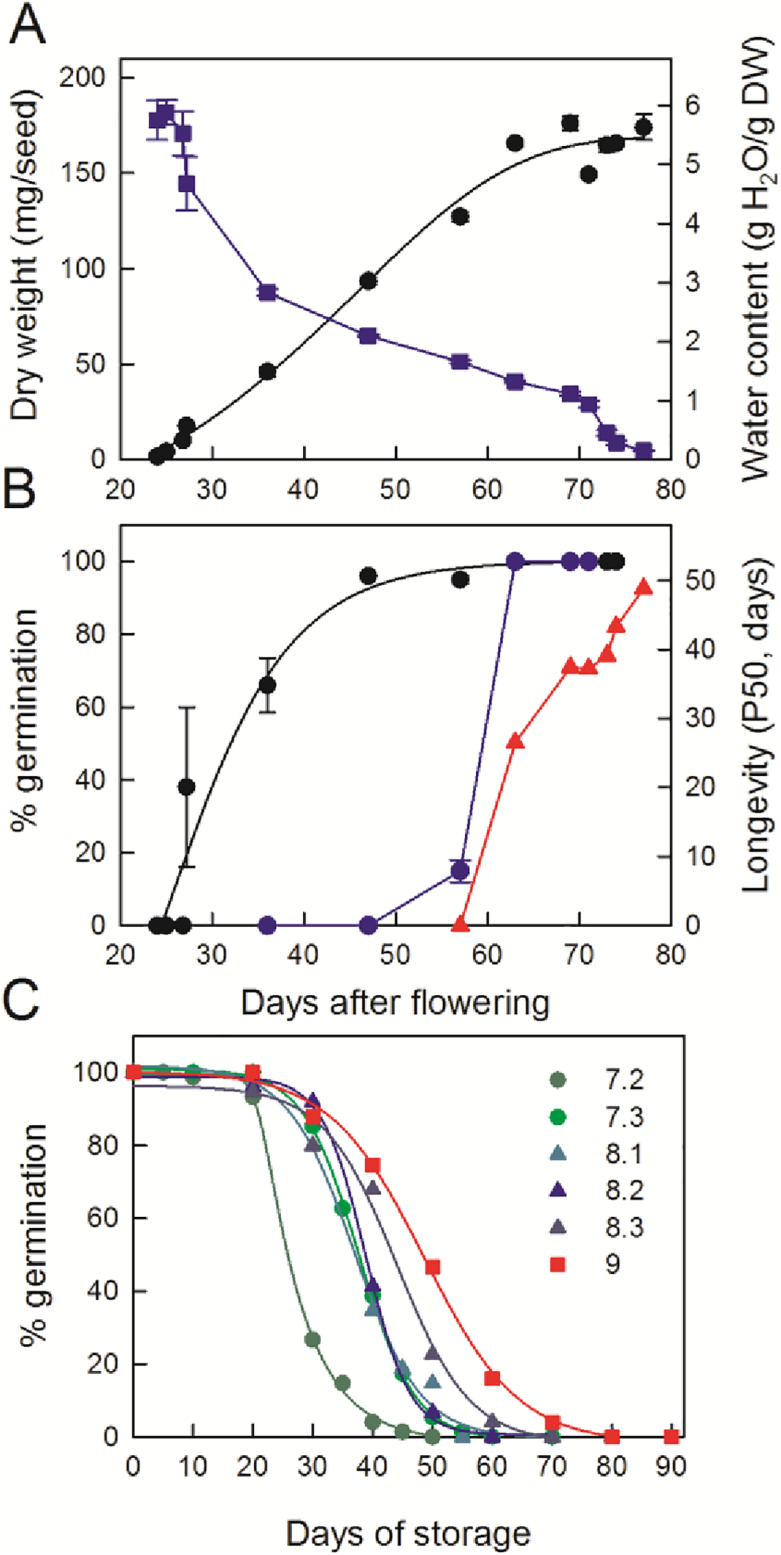
Physiological characterization of soybean seed maturation. (A) Evolution of seed dry weight (back circle) and water content (blue square). Data are the means (± SE) of 3 to 5 replicates of 20 seeds. (B) Acquisition of germinability (●, black circle) and desiccation tolerance (blue circle), evaluated after fast drying to 10% moisture and longevity (red triangle) as assessed by P50 (time necessary to obtain a loss of viability of 50% during storage 35°C and 75% RH). Data are the means (± SE) of 4 replicates of 25 seeds. (C) Loss of seed germination during storage at 75% HR, 35°C. Data are the mean of 4 replicates of 25 seeds harvested at indicated phenological stages.

Various events related to seed maturation were characterized in two consecutive years between reproductive stages R5 and R9. Seeds were collected at similar phenological stages between the 2013 and 2014 cultures to allow for comparison. Since no major developmental differences were found between both crops (S1 Fig), only data from the 2014 crop are presented here. Fig 2A shows that the end of the seed filling phase occurred around 63 d after flowering (DAF, corresponding to stage 7.2), whereas the onset of maturation drying started at 71 DAF (stage 8.1). The end of the seed filling phase also coincided with the abscission of seeds from the mother plant. At 57 DAF (stage 7.1) and 63 DAF (stage 7.2), 88% and 6% of the seeds were still attached to the fruit, respectively. At stage 7.3, all seeds were detached from the mother plant. In agreement with earlier works [4, 25, 27], germination capacity was acquired early during seed filling, between 25 and 57 DAF (stage 7.1). Desiccation tolerance, i.e. the ability to germinate after fast drying to 10% moisture, was acquired between 57 and 63 DAF (Fig 2B). There was no significant difference in the acquisition of these traits between the 2013 and 2014 crops (S1 Fig). From the loss of viability curves during storage at 75% RH and 35°C (Fig 2C), the longevity, expressed as the time to obtain 50% germination after storage (P50), was calculated. Seed longevity was progressively acquired shortly after the seed filling phase. During maturation, P50 values increased sharply between 57 and 63 DAF (stage 7.2) from 0 to 28 d (Fig 2B). Thereafter, P50 increased almost two-fold during further maturation, reaching 48 d in mature dry seeds. Longevity data obtained for the 2013 crop followed a similar trend as those of 2014 (S1 Fig), with the P50 being on average 18% lower than the 2014 crop. Monitoring field humidity and temperature revealed that the 2014 crop grew under significant higher average temperatures compared to 2013 (26.5°C and 30.2°C for 2013 and 2014, respectively).

### Transcriptome profiling during late maturation highlights dynamic changes during the acquisition of longevity

To characterize transcriptome changes during the end of seed maturation, total RNA was extracted from six stages from whole seeds during development from stage 7.1 until stage 9. A replication of stage of 7.2 and 9 was nested in the experimental design to assess reproducibility of the data. After sequencing and assembly, RNAseq produced between 14 and 38 million reads per library, with all libraries having >90% reads mapping (Table 1).

**Table 1:** Mapping of single-end reads to the soybean genome.

DAF	Stage	F/D	# reads	# Mapped reads	% Mapped reads
**57**	7.1	F	25 673,031	24 112,015	93.9
**63**	7.2a rep 1	F	38 088,839	34 971,702	91.8
** **	7.2b rep 2	F	34 381,008	31 799,644	92.5
	7.2a rep1	D	14 259,082	12 881,662	90.3
	7.2b rep2	D	66 764,127	60 626,937	90.8
**69**	7.3	F	22 267,371	20 475,504	92.0
**71**	8.1	F	14 533,337	13 392,050	92.2
**73**	8.2	F	22 057,408	19 621,133	89.0
**77**	9a rep 1	F	28 824,583	26 457,357	91.8
	9b rep 2	F	23 064,604	21 097,336	91.5

# reads, number of reads following trimming of the libraries for quality

# mapped reads, number of reads that unambiguously mapped to the soybean genome. Percentages of mapped sequences are also indicated. DAF, days after flowering; Rep, replicates. F: Freshly harvested seeds; D: rapidly dried seeds

**Fig 3:**
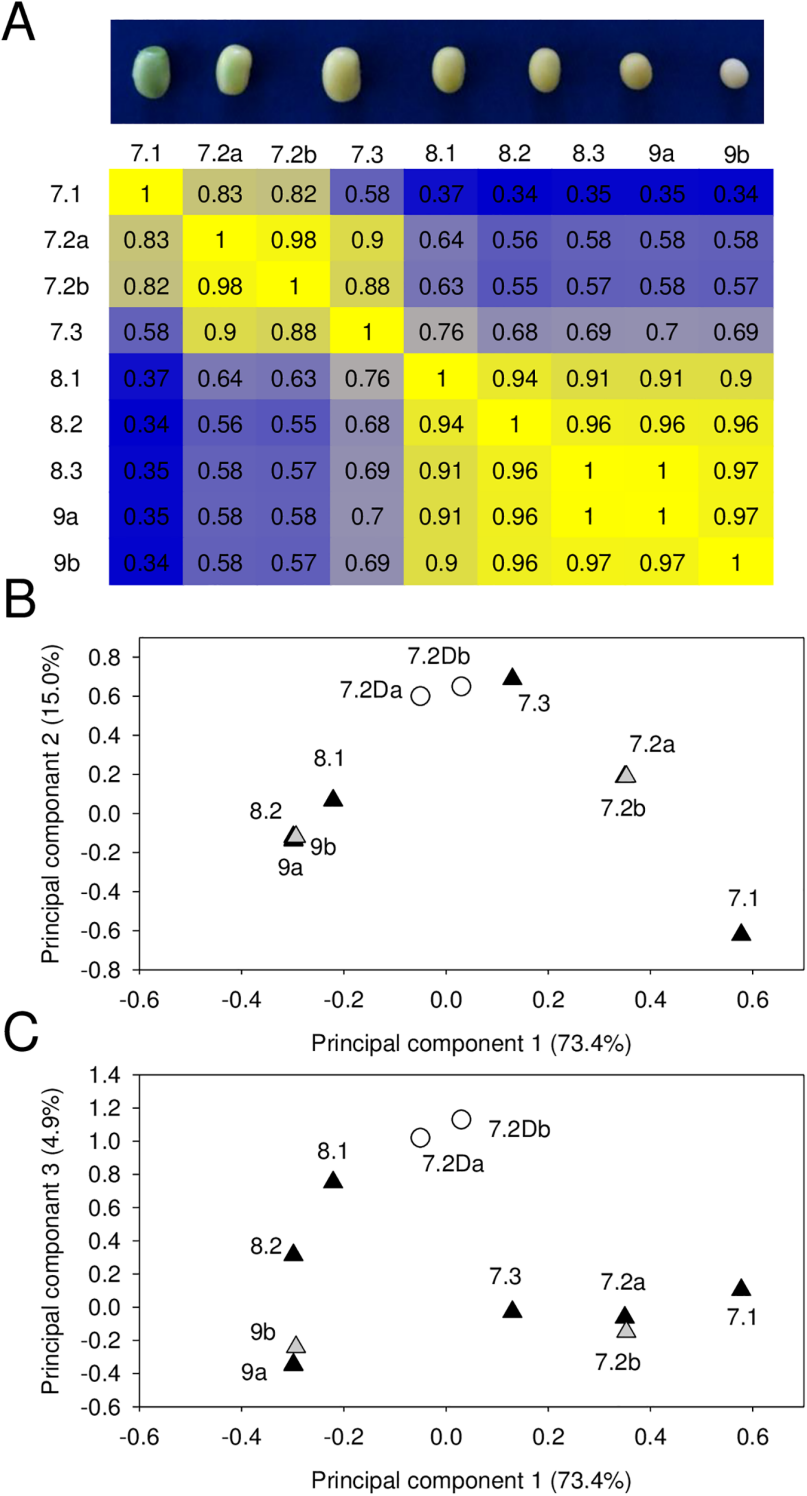
Correlation and principal component analyses of soybean transcriptomes during seed maturation. (A) Pair-wise Pearson correlation coefficients were used to generate the heat map. The color scale indicates the degree of correlation (blue, low; yellow, high). (B) and (C) Principal component analysis performed using median centering of the transcriptomes of seed phenological stages. 7.2D, transcriptomes of rapidly dried seeds at stage 7.2. The letters a,b, correspond to biological replicates.

From this dataset, 16,248 genes were retained as differentially expressed throughout the samples (*i*.*e*. with a variance > 1), S3 Table. PPC calculations and principal component analysis were performed to assess the dynamic response of the transcriptome throughout the maturation stages (Fig 3). Two transcriptional switches occurred during seed maturation, the first one between stage 7.1 and 7.2, visualized by the correlation matrix, and the second one between stage 7.3 and 8.1, apparent from the correlation matrix and the principal component analysis (Fig 3). Data from the two biological replicates were highly similar, with between 98% and 97% similarity between the two biological replicates of stage 7.2 and stage 9.

**Fig 4:**
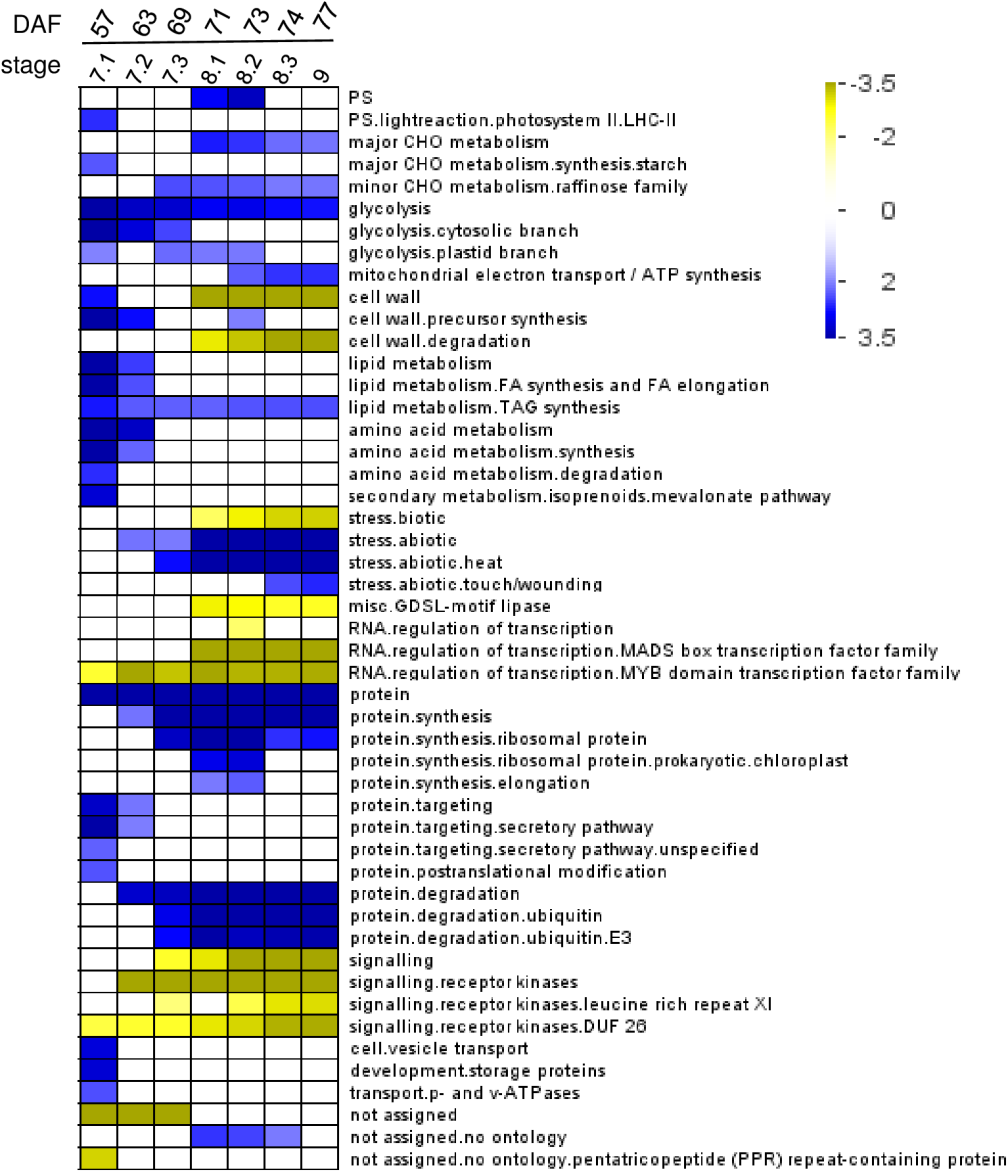
Over-representation analysis of functional classes during seed maturation. Functional classes and subclasses statistically affected are indicated according to Mapman ontology. Data were subjected to a Bin-wise Wilcoxon test and resulting p-values were adjusted according to Bonferroni. The scale bar indicates the z-score calculated from p-values (*i*.*e*. p-value of 0.05 represents a z-score of 1.96 after adjustment). Over-represented and under-represented classes are indicated respectively in blue and yellow.

Using Pageman (MAPMAN, GABI Germany), a gene ontology (GO) enrichment was performed to obtain an overview of the different biological processes that are overrepresented at the different stages of seed maturation (Fig 4). Stages 7.1 (and to a lesser extend stage 7.2) were characterized by an over-representation of functional classes related to growth and metabolic activities, reflecting the active seed filling that still went on in these green seeds. These classes included photosystem light reaction, starch synthesis, lipid metabolism and amino acid degradation, as well as storage proteins, protein targeting secretory pathway and cell vesicle transport. At stage 7.2–7.3, these classes were no longer over-represented, indicating the end of seed filling at the molecular level. From this developmental stage onwards, the class “abiotic stress” became overrepresented, as well as functional classes related to raffinose family sugars and protein degradation (ubiquitin), whereas classes corresponding to “signaling” became underrepresented. At stage 8.1, a transition occurred with a temporary overrepresentation of protein synthesis (elongation and ribosomal protein synthesis) and an underrepresentation of cell wall degradation and biotic stress. From stage 8.2 onwards, functional classes that became overrepresented were related to mitochondrial electron transport/ATP synthesis and abiotic stress (wounding/touch) (Fig 4).

### Accumulation of RFO in relation to the acquisition of longevity

**Fig 5:**
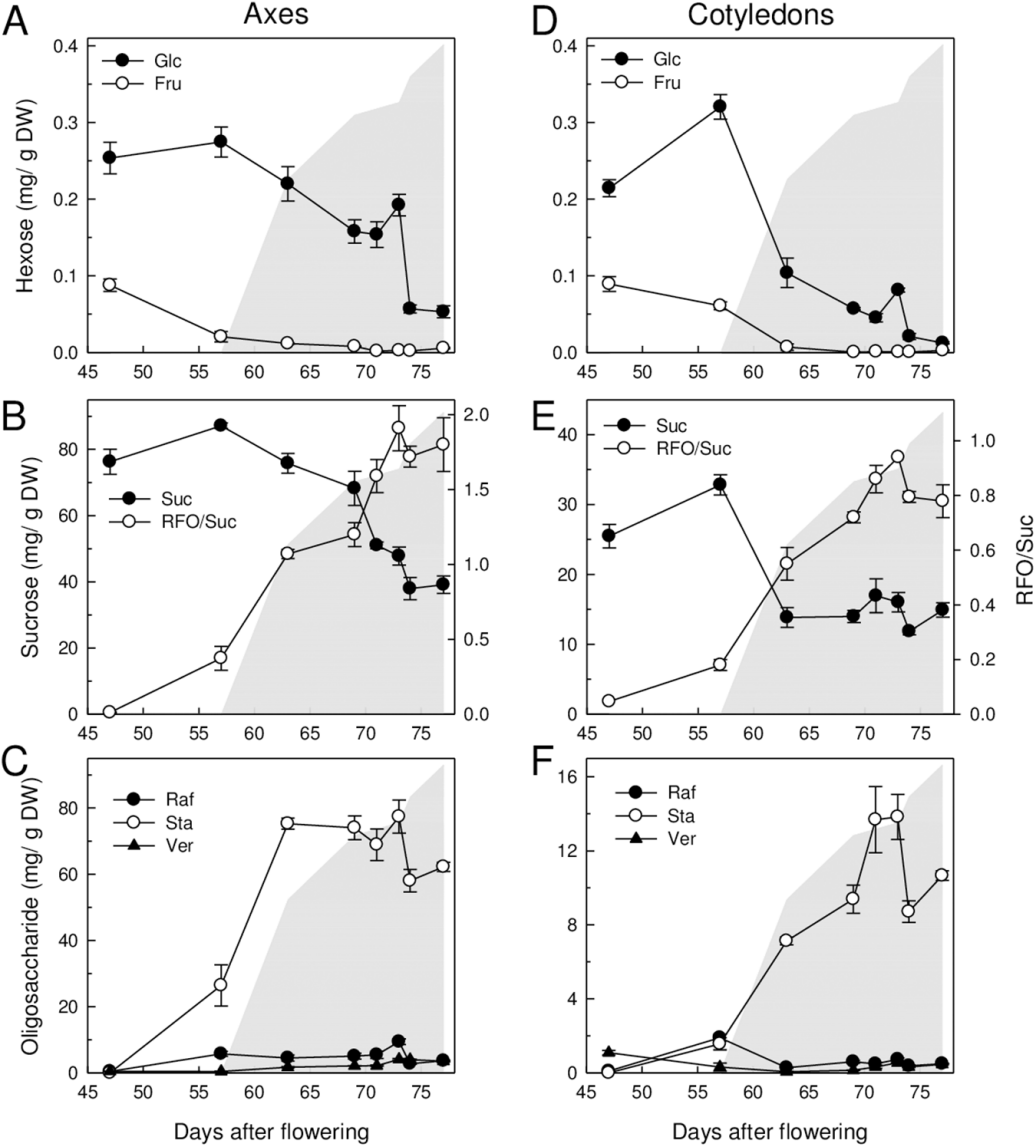
Changes in soluble sugar contents in axis and cotyledons during maturation. Data are the average of triplicates (± SE) using 6 axis (A-C) and 6 cotyledon pairs (D-E) from the 2014 crop. The changes in RFO/Suc ratio are shown in panel B and E for axes and cotyledons, respectively. The increase in longevity (P50) is indicated as a grey area as a help to the eye. (A, D) Glc, glucose and Fru, fructose; (B, E) Suc, sucrose and RFO/Suc ratio; (C, F) Raf, Raffinose, Sta, Stachyose and Ver, Verbascose.

One of the major changes occurring during seed maturation is the accumulation of RFOs. Numerous studies exist on the accumulation of these sugars during soybean seed development [42–44], but how this increase relates to the acquisition of seed longevity is unknown. The transcriptome analysis identified an overrepresentation of genes involved in RFO metabolism during maturation (Fig 4). Considering their controversial role in the survival in the dry state [10], we investigated the accumulation of the different soluble sugars in the axes and cotyledons during seed maturation and assessed whether these metabolic changes occurred in relation to the acquisition of longevity (Fig 5). Both in axes and cotyledons, glucose (Glc) and fructose (Fru) contents were highest during the seed filling phase and decreased throughout further maturation to almost undetectable levels. For Glu, this decrease occurred earlier in cotyledons compared to axes (Fig 5A and 5D). In axes, Suc contents remained high during the seed filling phase at around 80 mg/g DW (Fig 5B). At 57 DAF, Suc started to decrease until 73 DAF, when the seed moisture was 28% (DW basis). In cotyledons, Suc levels exhibited a sharp decrease at the end of the seed filling phase from 33 mg/DW at 57 DAF to 13 mg/g DW at 63 DAF (stage 7.2), concomitant with the decrease in Glu content and increase in longevity. Thereafter, Suc content remained constant until the dry state, representing 55% of the total amount of soluble sugars in the cotyledons. Stachyose (Sta) was the preponderant RFO in dry seeds, representing 90% of the total amount of RFO. In axis, its pattern of accumulation coincided with the acquisition of desiccation tolerance rather than longevity (Fig 5C). Sta accumulated during seed filling between 46 and 63 DAF then remained at steady level. In cotyledons, Sta contents increased later during maturation than in axis, along with the increase in longevity until 73 DAF (Fig 5F). Thereafter it decreased by *ca*. 30% during further maturation drying. The RFO/Suc ratio in the axis increased concomitantly with the increase in longevity (Fig 5B). By comparing Fig 5B and 5C, two successive factors contributed to this increase; first the synthesis of Sta (up to stage 7.3) then a decrease in Suc contents. In the cotyledons, the increase in RFO/Suc was mainly driven by the synthesis of RFO. Thus, the RFO/Suc ratio, especially in the embryonic axis, seems to be a good indicator of the seed longevity acquisition during maturation in this indeterminate cultivar.

### Identification of the transcription factor network involved in late seed maturation

**Fig 6:**
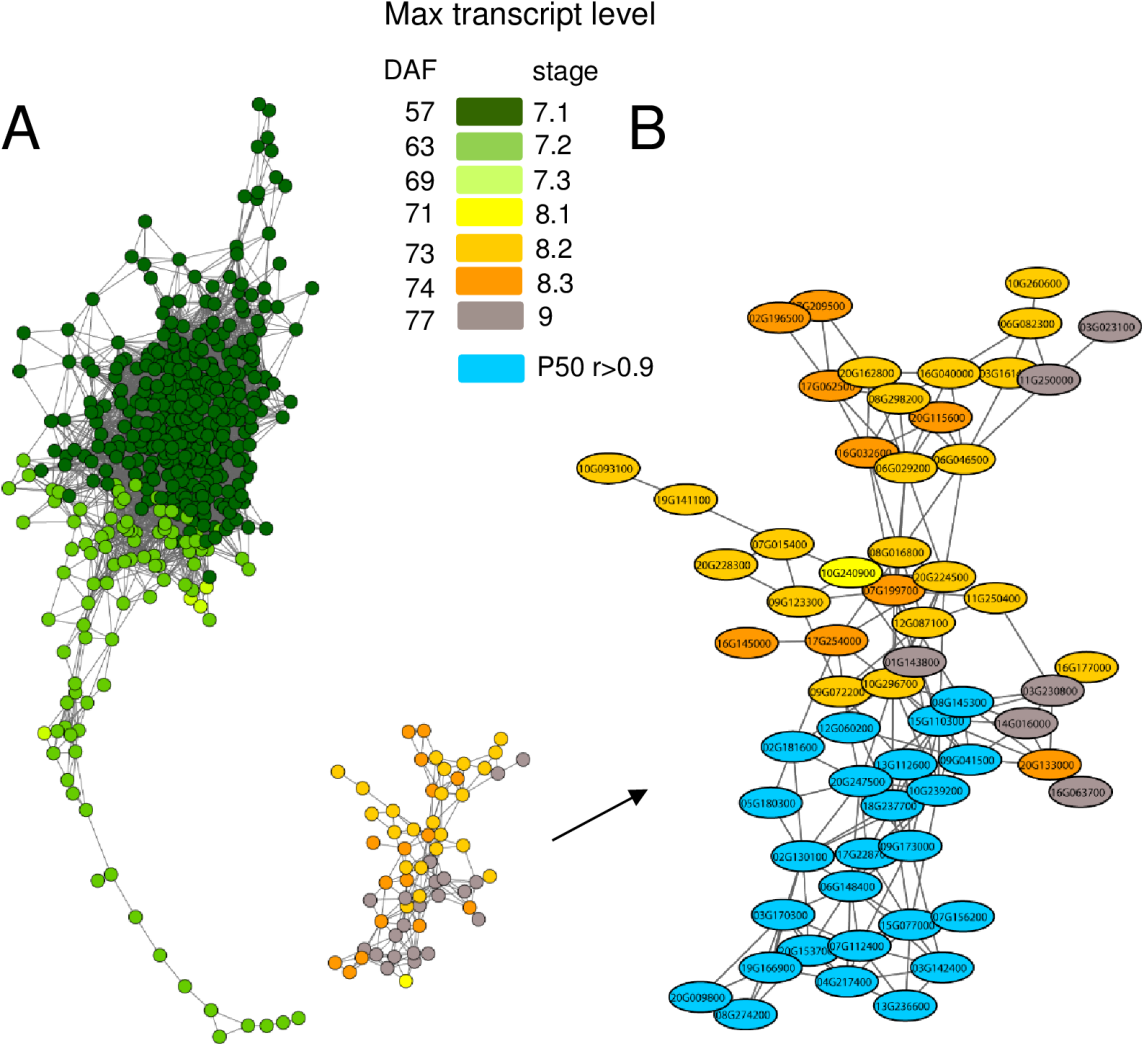
Transcription factor co-expression network of soybean seed maturation. (A) Gene co-expression network of seed maturation visualized using an organic layout in Cytoscape. Temporal analysis of nodes in the network was obtained by coloring each gene by its specific expression profile during seed development. Dark-green; high transcript levels at seed filling (stage7.1); green, light-green and yellow, transitory transcript levels maximum at stage 7.2, 7.3 and 8.1 respectively; light-orange, dark-orange and grey, transcript levels increasing during late seed development, being maximum at stage 8.2, 8.3 and stage 9. (B) Zoom on the gene module corresponding to late maturation. Nodes correlating with longevity (PCC>0.9) are colored in blue. Text labels indicate gene numbers.

To obtain information on the regulatory factors underlying the transcriptional and biochemical changes during maturation, changes in transcript profiles of genes encoding TF, representing 1086 genes belonging to 32 families, were further analyzed. To capture the temporal regulation of the TF transcripts during maturation, a gene co-expression network was constructed. The resulting scale-free network contained 499 nodes and 12,183 edges and was visualized using the cytoscape organic layout algorithm (Fig 6A). To understand the topology of the network, nodes were colored based on their expression profile, with the stage at which transcript levels were maximum. The network consisted of a central cluster of interacting nodes corresponding to genes with highest transcript level at the end of seed filling (Fig 6A, stage 7.1, dark green), followed by a tail composed of probes corresponding to TF with highest transcript level during the transition between seed filling and maturation drying phase (stage 7.2, light green). A second cluster that was disconnected from the main cluster represented TF with transcripts being maximum at stage 8.2 (yellow), 8.3 (orange) and 9 (mature seeds, grey).

Twenty four families of TF were present in the network (S4 Table, Fig 6A). Using a χ^2^ testing the null hypothesis that these families are randomly enriched in the different stages, AP2/EREBP and WRKY were the only families that were significantly enriched in the tail with genes exhibiting a transient expression profile with a maximum level around stage 7.2 and 7.3. At these stages, AP2/EREBP and WRKY represented 20% (p = 0.005) and 11% (p = 0.048) of all TF found differentially expressed, respectively. A closer inspection of these TF revealed genes involved in germination and organ growth, flowering, chloroplast dismantling, defense and ABA signaling.

**Fig 7:**
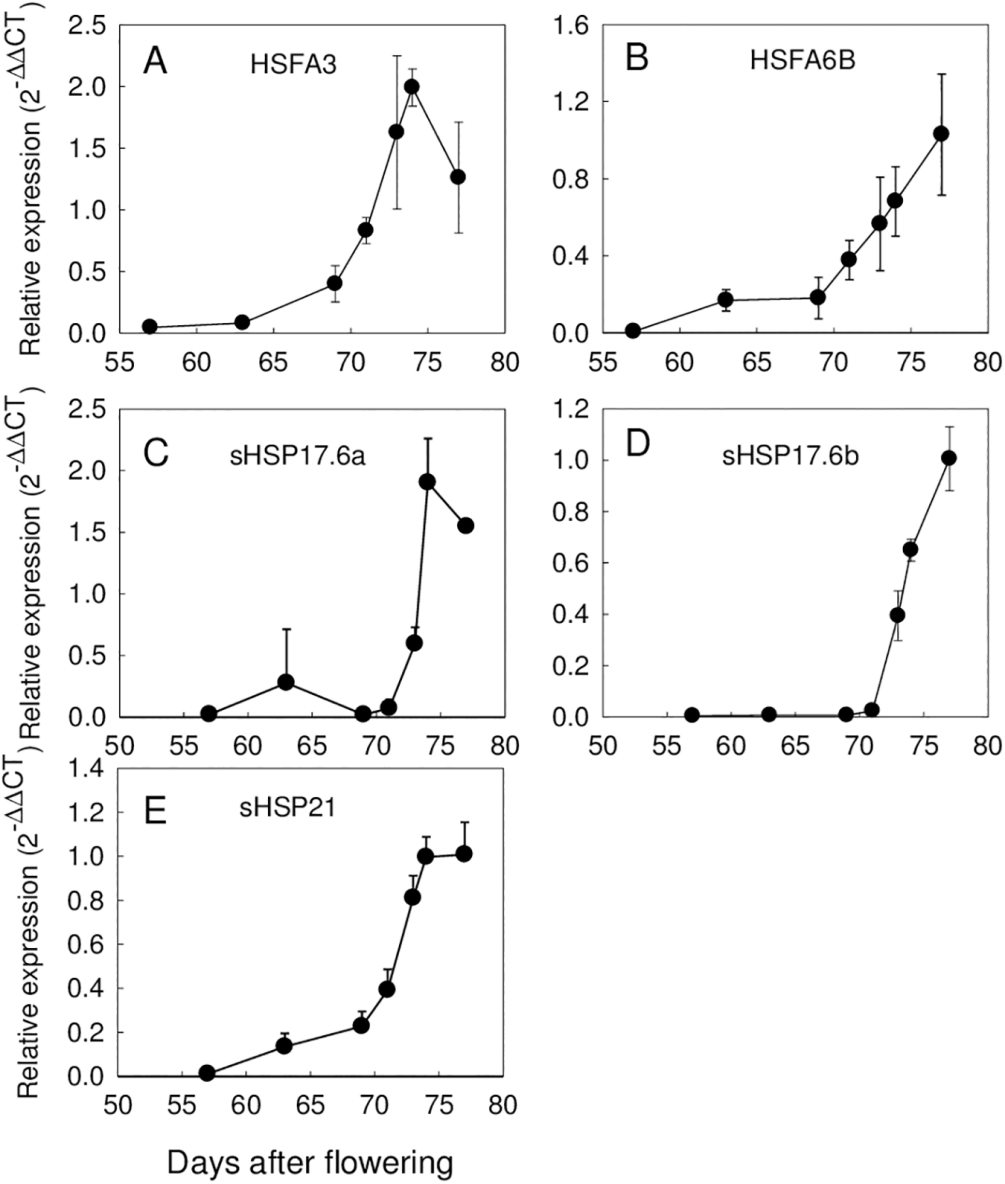
qPCR analysis of selected genes during seed maturation validates RNA-Seq data. (A) Heat shock transcription factor A3 (HSFA3: Glyma.03g191100); (B) Heat shock transcription factor A6B (HSFA6B: Glyma.03g157300); (C) sHSP17a (Glyma.17g224900); (D) sHPS17b (Glyma.14g099900), (E) sHSP21 (Glyma.08g318900). Data (±SE) are the average of three biological replicates of 30 seeds.

**Fig 8:**
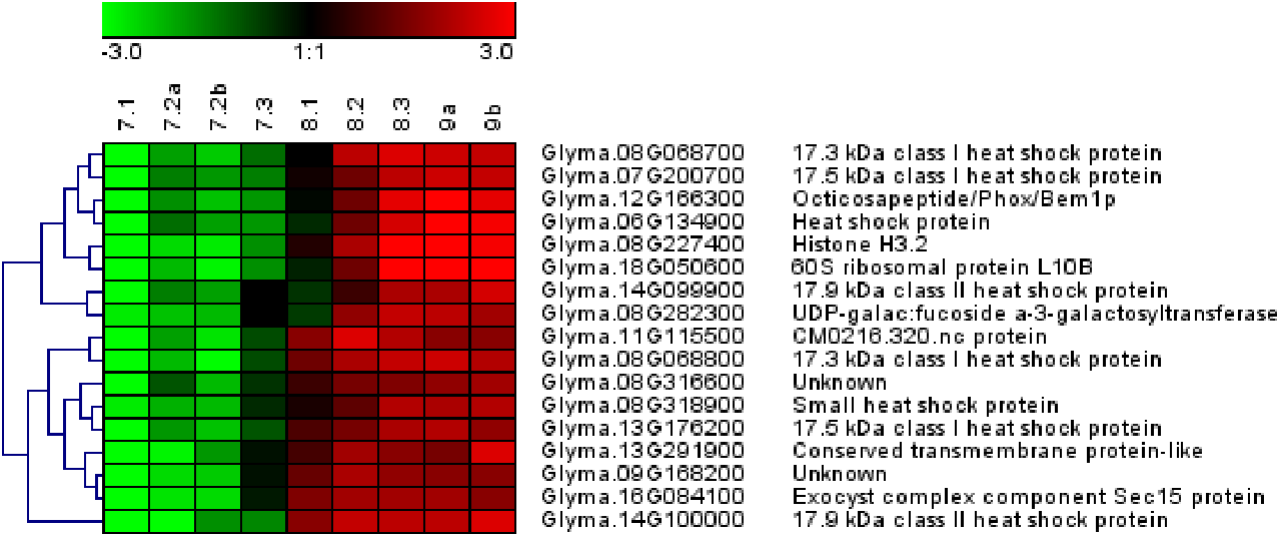
Heat map of most differentially expressed genes correlating with longevity. Genes were retained when the log2 intensity > 4 between stage 7.2 and 9 and that correlate with longevity (PPC P50>0.85). Genes were log2 mean centered and colored from the lowest (green) to highest values (red).

Next, we used the trait-based gene significance measure [38] to integrate the acquisition of longevity in the TF gene co-expression network to identify those TF that exhibit transcription profiles highly correlating with P50. For this purpose, PCC were calculated between the transcript levels of all TF genes and the P50 value during maturation (S4 Table). When genes with a PCC>0.9 were projected on the TF network, 27 TFs were identified that formed a highly co-expressed module (blue nodes Fig 6B, S4 Table). A total of 8 of the 27 genes belonged to the AP2/EREBP family, including four homologues of ETHYLENE RESPONSE FACTOR (ERF10, a member of the ERF subfamily B4, PCC>0.98) and homologues of the Arabidopsis DREB2C and DREB2F. Other TF belonged to families such as WRKY, auxin response factor, homeodomain-like proteins (S4 Table) and X-box binding transcriptional repressor family with a homologue of NUCLEAR TRANSCRIPTION FACTOR X-BOX BINDING LIKE 1 (*NFXL1*), a gene previously found in the longevity modules of *M*. *truncatula* and Arabidopsis co-expression network during seed maturation [24]. In addition, two heat shock factors (HSF, Glyma.03g191100 and Glyma.03g157300) were present in the list of the 27 TFs. RT-qPCR on additional seed samples from different developmental stages validated their increased transcript level during maturation (Fig 7A and 7B), with expression profiles being comparable to those obtained by RNAseq (r^2^>0.8). Interestingly, inspection of the Soybase gene expression profile showed that the paralogs of these two genes are not expressed in seeds. These HSF are transcriptional regulators known to activate small heat shock proteins (HSP). Consistent with this, analysis of the most differentially expressed genes between stage 7.2 and stage 9 (Int(log2)>4) that correlated with longevity (PPC>0.85) shows that many of these genes code for small HSP (Fig 8). RT-qPCR data confirmed the increase of transcript levels during final maturation for three sHSPs (Fig 7C–7E).

### Comparative analysis of dried, immature short-lived seeds with mature long-lived seeds identifies developmental indicators linked to longevity

Premature drying of immature seeds (stage 7.2) leads to reduced acquisition of longevity compared to fully mature seeds (Fig 2). A comparison of the transcriptome of these immature seeds with fully mature seeds can be used for further investigation of markers for longevity. First, the impact of enforced drying on the transcriptome of seeds harvested at stage 7.2 was visualized by PCA analysis (Fig 3). In comparison with the freshly harvested immature seeds, the transcriptome of the dried immature seeds was positioned between those of seeds harvested at stage 7.3 and 8.1 in the PCA plot (Fig 3). Apparently, premature drying accelerated the maturation of the seeds: *in planta*, the time lapse between stage 7.2 and 7.3 was 6–7 days (Fig 1), whereas it took only two days to dry the seeds harvested at stage 7.2.

**Fig 9:**
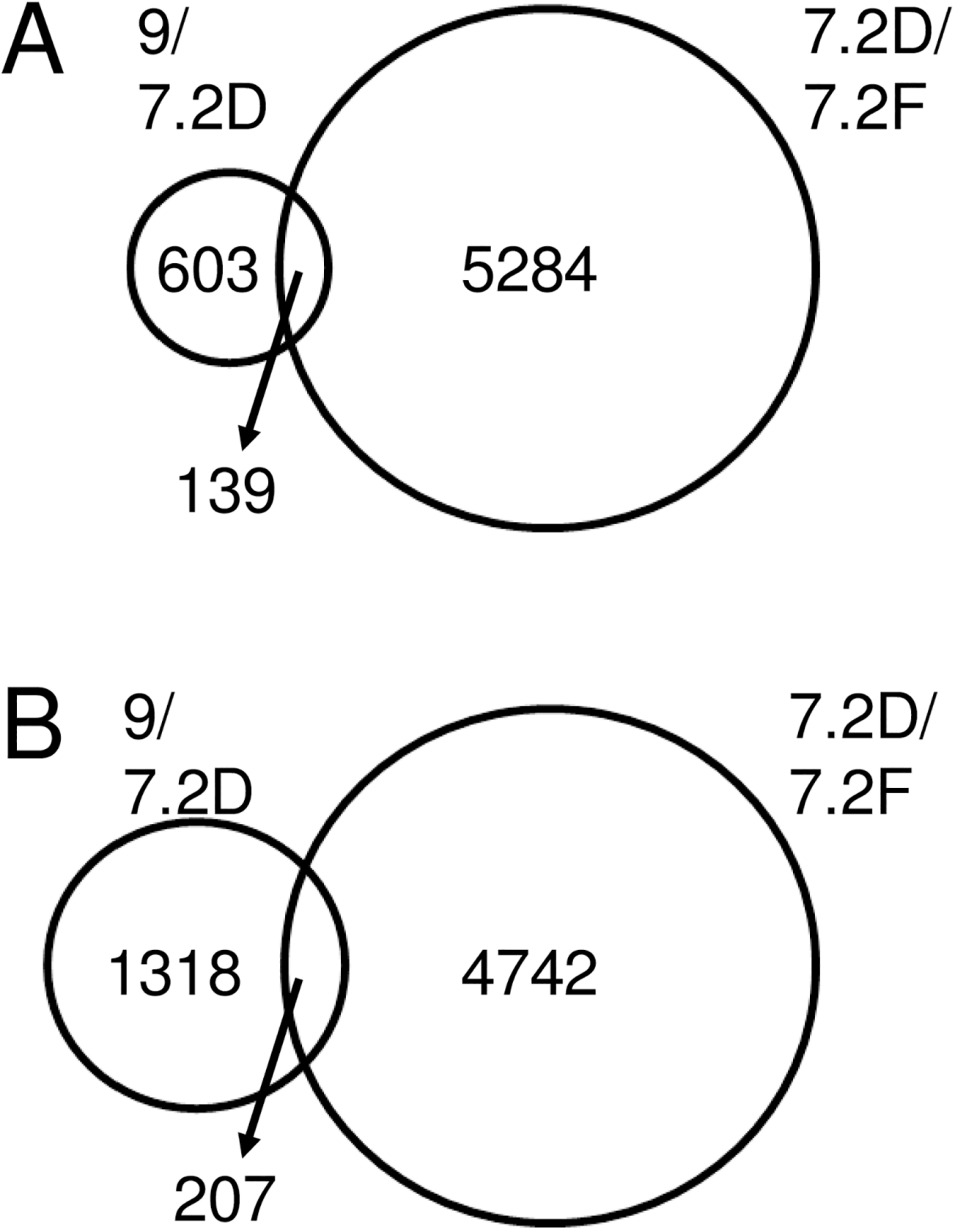
Venn diagrams identify transcripts correlating with longevity. Venn diagrams comparing transcripts that are differentially expressed in immature seeds that are rapidly dried at stage 7.2 (7.2D) compared to dry, mature seeds (stage 9) and transcripts that are differentially expressed between freshly harvested seeds at stage 7.2 (7.2F) and rapidly dried seeds at stage 7.2 (7.2D). Genes were considered statistically different when the absolute ratio was at least two fold with a P(BH)<0.01. (A). Number of genes with higher transcript levels in 9 vs 7.2D. (B). Number of genes with lower transcript levels in 9 compared to 7.2 D.

Transcriptome comparison between immature, rapidly dried seeds and mature seeds identified 742 genes with transcripts that were significantly higher in seeds with high longevity compared to those with low longevity, whereas 1525 genes had transcripts that were lower in the seeds with high longevity (S5 Table). Next, we investigated how many of these transcripts also changed during enforced drying. For this purpose, a statistical analysis was performed to identify the differentially expressed genes in seeds of stage 7.2 before and after enforced drying (S5 Table). A total of 5423 transcripts were up-regulated and 4931 down-regulated upon enforced drying of the seeds. The Venn diagram (Fig 9) shows that out of the 742 genes with higher transcript level at stage 9 compared to stage 7.2D, 139 (19%) also increased significantly during enforced drying. For the down-regulated transcripts, 207 out of the 1525 (14%) also responded to the enforced drying of the immature seeds (Fig 9, S5 Table).

A Gene Ontology (GO) enrichment analysis of the 139 differentially expressed genes with higher transcript level in both dried immature seeds and dry mature seeds revealed an overrepresentation of biological functions related to response to heat, hydrogen peroxide and high light intensity (Table 2). A closer look at the expression profiles of these genes showed that they increased sharply between stage 7.2 and stage 8.1, with a further gradual increase until stage 9 (S3 Table). Considering that enforced drying resulted in a transcriptome that was comparable to stages 7.3–8.1, the increased transcript levels between 7.2F and 7.2D could be the result of an accelerated maturation.

**Table 2:** GO enrichment analysis of the 139 differentially expressed transcripts that are significantly higher at stage 9 compared to 7.2 after drying and common between 7.2D/7.2F and 9/7.2D.

GO term	GO description	Genome GO count	Expressed GO	Corrected P-value
***Overrepresented***				
**GO:0009408**	response to heat	659	36	2.43E-22
**GO:0042542**	response to hydrogen peroxide	511	29	3.13E-18
**GO:0009644**	response to high light intensity	582	29	1.06E-16
**GO:0006457**	protein folding	779	29	2.24E-13
**GO:0034976**	response to endoplasmic reticulum stress	483	18	6.19E-08

Analysis was performed using the GO enrichment tool of Soybase using the Glyma 2.0 gene models.

An analysis of the enrichment of biological functions of the 603 genes that were up-regulated during maturation drying *in planta* but not upon enforced drying of freshly harvested seeds of stage 7.2 revealed functions related to pyrimidine ribonucleotide biosynthetic process, protein import into nucleus and protein targeting to mitochondrion, and nucleosome assembly (Table 3). Another biological function that was overrepresented is RNA methylation.

**Table 3:** GO enrichment analysis of the differentially expressed transcripts that are significantly higher at stage 9 compared to 7.2 after drying and not induced upon drying between 7.2F and 7.2D.

GO term	GO description	Genome GO count	Expressed GO	Corrected P-value
***Overrepresented***				
GO:0009220	pyrimidine ribonucleotide biosynthetic process	315	28	4.63E-21
GO:0006606	protein import into nucleus	243	23	2.69E-16
GO:0006626	protein targeting to mitochondrion	235	19	9.13E-15
GO:0006334	nucleosome assembly	128	12	2.62E-13
GO:0006164	purine nucleotide biosynthetic process	75	9	3.09E-08
GO:0001510	RNA methylation	418	22	9.26E-07
***Underrepresented***				
**GO:0006952**	defense response	1116	1	3.84E-05

Analysis was performed using the GO enrichment tool of Soybase using the Glyma 2.0 gene models.

Regarding the 207 genes that were down-regulated between stage 7.2D and stage 9 as well as stage 7.2F and stage 7.2D, no GO enrichment was found. In contrast, the enrichment analysis of differentially expressed genes with lower transcript levels in seeds with high longevity (1318) revealed an overrepresentation of biological functions related to response to generation of precursor metabolites, transcription, as well as cellular respiration (Table 4). In addition, biological processes to photosynthesis were down-regulated. A closer inspection of the list of genes belonging to these GO categories revealed that all of them were associated with various chloroplast functions such as photosynthesis and starch and lipid synthesis. Interestingly, the list also contained 69% of the soybean chloroplast genes (77 out 111, S5 Table), including those involved in the photosystem II reaction, ATP synthase sub-units, Calvin cycle (Rubisco large subunit), chlorophyll binding and translation (RNA polymerase, ribosomal proteins, maturase K). In addition, we found two homologues of STAY-GREEN1; an important gene involved in chlorophyll catabolism and photosystem degradation [45]. A large number of these genes were strongly upregulated in seeds of stage 7.2 that were submitted to enforced drying compared to freshly harvested seeds (S5 Table). Considering the acceleration of maturation in the dried seeds, this increase can be explained in part by their transitory expression profile, with transcript levels being higher in seeds at stage 8.1 compared to stage 7.2 (S3 Table).

**Table 4:** GO enrichment analysis of the 1318 differentially expressed transcripts that are significantly lower at stage 9 compared to prematurely dried seeds at stage 7.2.

GO term	GO description	Genome GO count	Expressed GO	Corrected P-value
**Overrepresented**				
**GO:0006091**	generation of precursor metabolites and energy	116	75	4.86E-82
**GO:0006354**	DNA-dependent transcription, elongation	225	75	4.58E-54
**GO:0015979**	Photosynthesis	452	89	3.33E-43
**GO:0019684**	photosynthesis, light reaction	320	42	6.04E-13
**GO:0045333**	cellular respiration	53	15	1.71E-08
**GO:0009772**	photosynthetic electron transport in photosystem II	28	11	1.47E-07
**GO:0009414**	response to water deprivation	888	62	5.35E-07
***Underrepresented***				
**GO:0009909**	regulation of flower development	744	1	8.88E-06
**GO:0006468**	protein phosphorylation	2386	32	3.65E-04

Analysis was performed using the GO enrichment tool of Soybase using the Glyma 2.0 Gene Models

Furthermore, an overrepresentation was found of processes related to water deprivation and salt stress (Table 4), which included genes involved in RFO metabolism such as galactinol synthase (Glyma.03g229800, Glyma.20g094500), raffinose synthase (Glyma.06g179200). In the GO term “response to water deprivation”; we noticed two Arabidopsis homologues of a RING domain ligase, namely RGLG1 and RGLG2, that have a ubiquitin E3 ligase activity and mediate the transcription of AtERF53 in response to drought [46].

## Discussion

Poor longevity results in economic losses due to the impossibility of carry-over of seed lots, having lost their vigor and viability so that they are no longer marketable. Identification of the underlying regulatory factors should provide information to design marker for prebreeding aiming to improve soybean seed quality. In developing soybean seeds, physiological maturity corresponds to the stage when final seed weight is reached, germination/desiccation tolerance and seed vigor are acquired [4, 25, 27]. In this study using an indeterminate cultivar, physiological maturity corresponded to stage 7.2, in agreement with previous works [4]. At this stage, most seeds were detached from the mother plant and had lost most of their chlorophyll. However, our physiological, sugar and transcriptome data show that the seed maturation program has not yet ended at physiological maturity. An additional period of 14 days after physiological maturity is necessary to acquire maximum longevity (Fig 2), in agreement with previous data on other genotypes [25, 26]. During this period, we detected 16,248 transcripts being differentially expressed until the developing seeds reached the dry state. Our RNAseq study complements and extends previous transcriptome characterization of soybean seed development [28–33]. These studies focused mostly from fertilization to end of seed filling whereas here we characterized the phase from end of seed filling to final maturation drying. Our RNAseq data and co-expression network analysis suggest that complex transcriptome changes occur after the so-called physiological maturity until the dry state, identifying several TFs associated with seed longevity. Several of these TFs were previously identified in a gene co-expression network associated with longevity in *M*. *truncatula* and Arabidopsis [24] and thus provide new resources for marker of seed development.

### Important transcriptional changes occur after mass maturity before the onset of desiccation

A salient feature of the genetic program that occurs during late seed maturation is a transitory phase that is delimited by two transcriptional switches, one from stage 7.1 to stage 7.2, marked by the end of seed filling, and the second between stages 7.3 and 8.1 (Fig 3). Phenotypically, this phase is accompanied by the loss of chlorophyll, acquisition of desiccation tolerance and the biggest increase in RFO sugars (Figs 2 and 6). A transcriptome shift was also observed in Arabidopsis during maturation and attributed to the desiccation of the seed [47]. This is not the case in soybean, since seed moisture content at stage 7.3 was around 1.2 g water/g DW, thus before the desiccation phase *per se*. However, during this transitory phase, seeds loose their connection to the mother plant. Concomitantly, changes in the transcriptome suggest the activation of a post-abscission program to prepare for the dry state and germination by synthesizing mRNA that will be stored until seed imbibition [48]. Several observations support this hypothesis. Genes with expression profiles that show maximum transcript levels at stage 7.2 or 7.3, i.e. represented by the tail of the co-expression network (Fig 6), are significantly enriched in members of the AP2/EREBP and WRKY families. AP2/EREBP transcription factors play an important role in controlling developmental processes and in hormone, sugar and redox signaling in relation with abiotic stresses [49]. Their over-representation in developing soybean seeds was also reported by Jones & Vodkin [32]. Several homologues were found to be co-regulated with the induction of longevity in the legume *M*. *truncatula* [23]. Most of the TFs were related to germination and growth, such as a homolog of WRKY6 (Glyma.13g310100) that in Arabidopsis acts as a positive regulator of ABA signaling during seed germination and early seedling development [50], the homolog of SOMNUS (Glyma.12g205700), the homolog of HOMEOBOX 1 (HB-1), involved in hypocotyl growth under short days [51] and a homolog of INDETERMINATE DOMAIN1/ENHYDROUS (Glyma.02g058500), that in Arabidopsis promotes the transition to germination by regulating light GA effects and ABA signaling during seed maturation [52]. We also found a B3 transcriptional repressor (Glyma.02g36090) whose Arabidopsis homologue is a negative regulator of seed size in developing seeds [53]. Transcripts associated with protein degradation via the SCF family of modular E3 ubiquitin pathway increased during maturation drying at stage 7.3. This observation is in agreement with previous studies [28, 32]. These proteins are known to filter the proteome by degrading key regulatory proteins as main targets [54]. This suggests that the drying seeds are setting up a machinery for post-translational regulation before entering in the dry state that will presumably serve upon imbibition.

### An emerging picture of longevity-related genes identifies confirmed regulators and newcomers in legume seeds

The network analysis revealed 27 TF whose transcript profiles are correlated with P50 (Fig 6, S4 Table), thereby forming a longevity module similar to that found in developing Arabidopsis and *M*. *truncatula* [23, 24]. Indeed, the homologues of 7 soybean genes were also present in the list of the 9 TF belonging to Medicago longevity module [24], namely three homologues of an ERF110 of Arabidopsis (Glyma.06g148400, Glyma.04g217400, Glyma.08g145300), a homologue of the ETHYLENE INSENSITIVE PROTEIN 3 family (Glyma.05g180300), two homologues of the DREB2 family (Glyma.07g156200, Glyma.14g056200), and a homologue of NF-X-like1 gene (Glyma.09g173000). The implication of two of these genes in longevity has been demonstrated [55, 24]. A DREB2 from sunflower enhanced seed longevity of tobacco when ectopically over-expressed with a heat shock factor, HaHSFA9 [55]. In Arabidopsis, seeds of *nfxl1* exhibited impaired acquisition of longevity during maturation [24]. When overexpressed in vegetative tissues, NFXL1 induces a higher survival upon salt stress, drought and high light intensity [56].

Several TF that highly correlated with P50 are associated with auxins and gibberellins (S3 and S4 Tables), such a homologue of HECATE2 (Glyma.11g055300), that affects auxin responses in Arabidopsis during flower development [57], a homologue of AUXIN RESPONSE FACTOR19 (ARF19, Glyma.13g112600, Glyma.09g072200), ARF4 (Glyma. 12G171000), ARF8 (Glyma.10G210600) ARF9 (Glyma.03g36710), ARF10 (Glyma.13g325200), ARF16 (Glyma.10g210600). This reinforces the link between auxin and longevity previously found in the Medicago and Arabidopsis maturation network, where 60% of genes belonging to the longevity were significantly enriched in binding sites for auxin response factor [24]. The putative role of auxins in inducing longevity remains to be investigated. These auxin-related genes exhibit functions that are associated with embryogenesis, meristem maintenance, stem cell specification, positioning of lateral organs and organ growth meristem in connection with GA signaling [57–59]. A further inspection of the TF present in the network tail (Fig 6) confirmed the enrichment in genes with the above mentioned functions such as the homologue of JACKDAW (Glyma.10g051500), AINTEGUMENTA-LIKE 6 (Glyma.01g022500), SEPALLA3 (Glyma.20g153700) and GATA (Glyma.17g228700). We speculate that the presence of these transcripts in dry seeds may be necessary to anticipate the restoration of the developmental fate of specific cells during germination of seeds that were damaged by ageing during storage. Consistent with this, stem cell niches are hypersensitive to DNA damage [60], which is a known cause for decrease in seed viability after storage [21].

The transcriptome comparison between immature short-lived and mature seeds exhibiting maximal life span further highlights key mechanisms that could be involved in longevity. Seed longevity correlated with the synthesis of HSPs and several chaperones implicated in protein-protein interactions and protein folding (Table 2, Fig 8, S5 Table). They are known to assist in creating proper-folding conditions during abiotic stress [61] and protect against oxidative stress during storage [62] that could be conducive to seed longevity. Our transcriptome also revealed the presence of HSFA6B (Glyma.03G157300), whose transcript profile was correlated with P50 (S4 Table). This is consistent with the observation that the over-expression of sunflower *HaHSFA9* in tobacco led to an increased stability against accelerated ageing of the seeds [13]. This TF also interacts with the drought-responsive factor HaDREB2 in a seed-specific manner to enhance stability against accelerated aging [55].

Concomitant with the acquisition of longevity, there was an overrepresentation of genes involved in RFO synthesis (Table 3). This was confirmed by the observation that RFO content and the ratio Suc/RFO increased during late seed maturation (Fig 5), reinforcing the idea that the regulation of RFO metabolism occurs at the transcriptional level [10, 12]. The role of RFO in seeds is unclear, particularly in soybean. No defects in desiccation tolerance, seed germination or seedling emergence were reported in soybean lines with low RFO content [44, 63]. However, recent literature suggests that RFO metabolism plays a role in the acquisition of longevity. Galactinol, the precursor of RFO was found to be a marker for seed longevity in Arabidopsis, cabbage and tomato [64]. In Arabidopsis, seeds of galactinol synthase mutants (*gols2* and *gols1gols2)* were more sensitive to accelerated aging (85% RH, 40°C) while seeds of *raffinose synthase* (*rs*) and *stachyose synthase* (*sts*) mutants did not differ significantly from those of wild type [64]. Further genetic proof for a role for RFO metabolism in longevity came from the impaired shelf-life of Arabidopsis *α-galactosidase2* (*agal2*) seeds stored at 75% RH and 35°C [24]. Suc levels were significantly higher in these mutants but no change was detected in RFO levels. Seed-specific overexpression of *CaGolS1* and *CaGolS2* of chickpea in Arabidopsis resulted in an improved resistance against controlled deterioration [11]. However, the moisture content of these seeds during ageing was 24%, a value high enough to lead to increased metabolic activity and accumulation of RFO, while this would not occur during dry storage at RH below 75%. Additional correlative evidence of a link of RFO metabolism and longevity comes from the analysis of mutants of two regulatory genes of seed maturation in *M*. *truncatula*: Mt-*abi5* and Mt-*snf4b* [12, 35]. Seeds of these mutants show decreased RFO accumulation and increased Suc accumulation during maturation together with a decreased in longevity. The underlying mechanisms that explain the role of galactinol or RFO in conferring longevity remain elusive. A specific role of RFO in the protection of membranes or other macromolecules seems unlikely. Suc molecules are known to protect membranes just as efficiently as RFO and furthermore make denser glasses (reviewed in [10]). Some studies suggest a direct or indirect role of RFOs in the protection against oxidative damage during storage [11, 65]. Overall, our data show that the RFO/Suc ratio in the embryonic axis is a good indicator of the progress in the acquisition of longevity during maturation. However, one should keep in mind that although this relationship is valid during seed development, it might be blurred when comparing mature seeds from different genotypes, environments and date of planting.

Typically, late seed maturation is characterized by a degreening process resulting from the degradation of chlorophyll via a multi-step catabolic pathway that is characterized mostly during leaf senescence [41, 45]. Here, chlorophyll was lost during the transition phase, between stage 7.1 and 7.3. Genes encoding enzymes that are required for the initiation of the degradation of chlorophyll and light harvesting complexes such as chlorophyll b reductase (NYC1, Glyma.07g085700, Glyma.09g191200) and STAYGREEN1 (Glyma01g241600, Glyma011g027480) were already highly expressed at stage 7.1 and increased slightly at stage 7.2. This suggests that on a transcriptional level, the onset of degreening is activated prior to the induction of longevity. Furthermore, a high number of transcripts associated with photosynthesis and chloroplast activity were down-regulated when artificially dried immature seeds at stage 7.2 were compared with mature dry seeds at stage 9 (Table 4). This decrease involved both nuclear and chloroplastic genes among which 69% of the genome was concerned. It might reflect a major shutdown of chloroplast metabolism and dismantlement, which requires a close coordination between the nucleus and chloroplastic genomes. These events might be conducive to seed longevity. The presence of green seeds in mature seed lots has been associated with a decreased shelf-life during storage in various species, including soybean [12, 22, 66]. In Arabidopsis, seeds of mutants affected in chlorophyll degradation contained 10-fold more chlorophyll than the wild type and had a strongly reduced longevity [22]. Chlorophyll degradation and longevity were affected during maturation of pea and *M*. *truncatula* seeds defective in ABCSISIC ACID INSENSITIVE5 [12]. The more chlorophyll was retained in mature in *abi5* seeds, the more longevity was affected. In addition, the repression in photosynthesis-associated genes during maturation was also impaired in these *abi5* mutants [12]. Thus, these data reinforce the idea that degreening and chloroplast dismantling appears a pre-requisite for longevity.

In this study, the storage conditions were set at a RH of 75% and a temperature of 35°C that bring the seed tissues to a water content of 0.13 g H_2_O/g DW, equivalent to 11.5% on a fresh weight basis. In commercial practice, soybean seeds are harvested at 12–14% moisture to avoid mechanical damage and dried to 10–12% for short-term storage [4]. Drying to lower values is known to make the seeds more susceptible to cracking. The combination 75%RH/35°C also represents a good compromise between conditions that are deleterious enough to induce a loss of viability to allow measurements within a reasonable experimental time but low enough to be near the glassy state where metabolism no longer occurs. Whereas a storage environment of 75% RH/35°C brings the cytoplasm out of the glassy state, it is still in an amorphous rubber with solid-like properties [7, 8, 67, 68]. It has been demonstrated that measurements of longevity in more humid conditions (i.e. ≥ 85% RH) are unreliable to predict life span in storage conditions corresponding to the dry state [68–70]. In soybean, this RH will bring the water content of the tissues around 0.24 g H_2_O/g DW (19% FW basis). Under these conditions, the cytoplasm will no longer be in a rubbery state, where molecular movement will still be restricted, but rather in a liquid state, allowing for metabolism to occur [7, 8, 67]. Quantitative trait loci analysis of seed aging in *M*. *truncatula* at 60%/35°C and 75% RH/ 35°C revealed similar loci and data between both aging conditions were very well correlated (r = 0.71), suggesting similar mechanisms of deterioration between these two RH [12]. However, comparison of aging at 30% RH/9°C and 75% RH/50°C in different genotypes of lettuce showed poor correlation [70]. Thus, one should remain careful with extrapolating our soybean data to dry storage conditions (<50% RH). Indeed, in soybean water properties change cotyledons below 8% (DW basis), most likely due to the formation of a glassy state in the cells whereas the respiration rate becomes detectable at 24% [71].

## Supporting information

S1 Fig Comparison of soybean seed development for the 2013 and 2014 harvest at indicated phenological stages. (A) Evolution of seed dry weight (black circles) and water content (blue squares). Data are the means (± SE) of 3 to 5 replicates of 20 seeds. (B) Acquisition of germination (black circles) and desiccation tolerance (blue squares), evaluated after fast drying to 10% moisture. (C) Acquisition of longevity as assessed by P50 (time necessary to obtain a loss of viability of 50% during storage 35°C and 75% RH). Data are the means (± SE) of 4 replicates of 25 seeds. Data are presented for 2013 (open symbols) and 2014 (closed symbols).
(TIF)

S1 Table Description of the reproductive phenological stages of soybean seeds. (PDF)

S2 Table List of primers combination used for gene expression validation of target genes by qRT-PCR. (DOCX)

S3 Table List of differentially expressed genes during seed maturation. (XLSX)

S4 Table Transcription factors present in the co-expression network of soybean seed maturation. Genes with expression profiles that correlate with longevity (P50, PCC>0.9) are indicated in bold with a blue background.
(XLSX)

S5 Table List of differentially expressed genes associated with longevity. List of differentially expressed genes that are higher/lower in mature seeds exhibiting maximal longevity (stage 9) compared to dried immature seeds (stage 7.2D) with low longevity.
(XLSX)

## References

[1] Finch-Savage WE, Bassel GW. Seed vigour and crop establishment: extending performance beyond adaptation. J. Exp. Bot. 2016;67:567–91. doi: 10.1093/jxb/erv490 26585226 10.1093/jxb/erv490

[2] Walters C, Wheeler LM, Grotenhuis JM. Longevity of seeds stored in a genebank; species characteristics. Seed Sci. Res. 2005;15:1–20. doi: 10.1079/SSR2004195

[3] Kueneman EA. Genetic control of seed longevity in soybeans. Crop Sci. 1983;23 5–8.

[4] Marcos-Filho J. Seed physiology of cultivated plants 2^nd^ Edition Londrina: Associação Brasileira de Tecnologia de Sementes-ABRATES; 2016

[5] Li DZ, Pritchard HW. The science and economics of ex situ plant conservation. Trends Plant Sci. 2009;14:614–21. doi: 10.1016/j.tplants.2009.09.005 19818672 10.1016/j.tplants.2009.09.005

[6] Hay FR, Probert RJ. Advances in seed conservation of wild plant species: a review of recent research. Conserv. Physio. 2013;1:cot030 doi: 10.1093/conphys/cot030 27293614 10.1093/conphys/cot030 PMC4806614

[7] Walters C, Ballesteros D, Vertucci VA. Structural mechanics of seed deterioration: standing the test of time. Plant Sci. 2010; 179:565–73. doi: 10.1016/j.plantsci.2010.06.016

[8] Buitink J, Leprince O, Hemminga MA, Hoekstra FA. Molecular mobility in the cytoplasm: An approach to describe and predict lifespan of dry germplasm. Proc. Natl. Acad. Sci. USA. 2000; 97:2385–90. doi: 10.1073/pnas.040554797 10681458 10.1073/pnas.040554797 PMC15810

[9] Sano N, Raijou L, North NM, Debeaujon I, Marion-Poll A. Seo M. Staying alive: molecular aspects of seed longevity. Plant Cell Physiol. 2016; 57:660–74. doi: 10.1093/pcp/pcv186 26637538 10.1093/pcp/pcv186

[10] Leprince O, Pellizzaro A, Berriri S, Buitink J. Late seed maturation: drying without dying. J. Exp. Bot. 2017;68:827–41. doi: 10.1093/jxb/erw363 28391329 10.1093/jxb/erw363

[11] Salvi P, Saxena SC, Petla BP, Kamble NU, Kaur H, Verma P et al Differentially expressed galactinol synthase(s) in chickpea are implicated in seed vigor and longevity by limiting the age induced ROS accumulation. Sci. Rep. 2016;6:35088 doi: 10.1038/srep35088 27725707 10.1038/srep35088 PMC5057127

[12] Zinsmeister J, Lalanne D, Terrasson E, Chatelain E, Vandecasteele C, Vu BL et al ABI5 is a regulator of seed maturation and longevity in legumes. Plant Cell 2016; 28:2735–54. doi: 10.1105/tpc.16.00470 27956585 10.1105/tpc.16.00470 PMC5155344

[13] Tejedor-Cano J, Prieto-Dapena P, Almoguera C, Carranco R, Hiratsu K, Ohme-Takagi M, et al Loss of function of the HSFA9 seed longevity program. Plant Cell Environ. 2010;33:1408–17. doi: 10.1111/j.1365-3040.2010.02159.x 20444218 10.1111/j.1365-3040.2010.02159.x

[14] Hundertmark M, Buitink J, Leprince O, Hincha DK. The reduction of seed-specific dehydrins reduces seed longevity in *Arabidopsis thaliana*. Seed Sci. Res. 2011;21:165–73. doi: 10.1017/S0960258511000079

[15] Chatelain E, Le Gall S, Hundertmark M, Leprince O, Satour P, Deligny-Penninck S, et al Temporal profiling of the heat stable proteome during late maturation of *Medicago truncatula* seeds identifies a restricted subset of late embryogenesis abundant proteins associated with longevity. Plant Cell Environ. 2012; 35:1440–55. doi: 10.1111/j.1365-3040.2012.02501.x 22380487 10.1111/j.1365-3040.2012.02501.x

[16] Nagel M, Kranner I, Neumann K, Rolletschek H, Seal CE, Colville L, et al Genome-wide association mapping and biochemical markers reveal that seed ageing and longevity are intricately affected by genetic background and developmental and environmental conditions in barley. Plant, Cell Environ. 2015;38:1011–22. doi: 10.1111/pce.12474 25328120 10.1111/pce.12474

[17] Sattler S E, Gilliland LU, Magallanes-Lundback M, Pollard M, DellaPenna D. Vitamin E is essential for seed longevity, and for preventing lipid peroxidation during germination. Plant Cell 2004;16:1419–32. doi: 10.1105/tpc.021360 15155886 10.1105/tpc.021360 PMC490036

[18] Debeaujon I, Leon-Kloosterziel KM, Koornneef M. Influence of the testa on seed dormancy, germination, and longevity in Arabidopsis. Plant Physiol 2000;122:403–13. doi: 10.1104/pp.122.2.403 10677433 10.1104/pp.122.2.403 PMC58877

[19] Boca S, Koestler F, Ksas B, Chevalier A, Leymarie J, Fekete A. et al Arabidopsis lipocalins AtCHL and AtTIL have distinct but overlapping functions essential for lipid protection and seed longevity. Plant Cell Environ. 2014;37:368–81. doi: 10.1111/pce.12159 23837879 10.1111/pce.12159

[20] Oge L, Bourdais G, Bove J, Collet B, Godin B, Granier F, et al Protein repair L-Isoaspartyl methyltransferase1 is involved in both seed longevity and germination vigor in Arabidopsis. Plant Cell 2008; 20:3022–37. doi: 10.1105/tpc.108.058479 19011119 10.1105/tpc.108.058479 PMC2613667

[21] Waterworth W, Masnavi G, Bhardwaj RM, Jiang Q, Bray CM, West CE. 2010. A plant DNA ligase is an important determinant of seed longevity. Plant J. 2010;63:848–60. doi: 10.1111/j.1365-313X.2010.04285.x 20584150 10.1111/j.1365-313X.2010.04285.x

[22] Nakajima S, Ito H, Tanaka R, Tanaka A. Chlorophyll b reductase plays an essential role in maturation and storability of Arabidopsis seeds. Plant Physiol. 2012;160:261–73. doi: 10.1104/pp.112.196881 22751379 10.1104/pp.112.196881 PMC3440204

[23] Verdier J, Lalanne D, Pelletier S, Torres-Jerez I, Righetti K, Bandyopadhyay K, et al A regulatory network-based approach dissects late maturation processes related to the acquisition of desiccation tolerance and longevity of *Medicago truncatula* seeds. Plant Physiol. 2013;163:757–74. doi: 10.1104/pp.113.222380 23929721 10.1104/pp.113.222380 PMC3793056

[24] Righetti K, Vu JL, Pelletier S, Vu BL, Glaab E, Lalanne D, et al Inference of longevity-related genes from a robust co-expression network of seed maturation identifies regulators linking seed storability to biotic defense-related pathways. Plant Cell 2015;27:2692–708. doi: 10.1105/tpc.15.00632 26410298 10.1105/tpc.15.00632 PMC4682330

[25] Zanakis GN, Ellis RH, Summerfield RJ. Seed quality in relation to seed development and maturation in 3 genotypes of soyabean (*Glycine max*). Expl. Agric. 1994;30:139–156.

[26] Gillen AM, Smith JR, Mengistu A, Bellaloui N. Effects of maturity and *Phomopsis longicolla* on germination and vigor of soybean seed of near-isogenic lines. Crop Sci. 2012;52:2757–66. doi: 10.2135/cropsci2011.10.0566

[27] Zanakis GN, Ellis RH, Summerfield RJ. A comparison of changes in vigour among three genotypes of soyabean (*Glycine max*) during seed development and maturation in three temperature regimes. Expl. Agric. 1994;30:157–70.

[28] Jones SI, Gonzalez DO, Vodkin LO. Flux of transcript patterns during soybean seed development. BMC Genomics 2010;11:136 doi: 10.1186/1471-2164-11-136 20181280 10.1186/1471-2164-11-136 PMC2846912

[29] Severin AJ, Woody JL, Bolon Y-T, Joseph B, Diers BW, Farmer AD, et al RNA-seq atlas of *Glycine max*: a guide to the soybean transcriptome. BMC Plant Biol. 2010;10:160 doi: 10.1186/1471-2229-10-160 20687943 10.1186/1471-2229-10-160 PMC3017786

[30] Asakura T, Tamura T, Terauchi K, Narikawa T, Yagasaki K, Ishimaru Y, et al (2012) Global gene expression profiles in developing soybean seeds. Plant Physiol Biochem. 2012;52:147–53. doi: 10.1016/j.plaphy.2011.12.007 22245912 10.1016/j.plaphy.2011.12.007

[31] Shamimuzzama M, Vodkin L. Identification of soybean seed developmental stage-specific and tissue-specific miRNA targets by degradome sequencing. BMC Genomics 2012;13:310 doi: 10.1186/1471-2164-13-310 22799740 10.1186/1471-2164-13-310 PMC3410764

[32] Jones SI, Vodkin LO. Using RNA-Seq to profile soybean seed development from fertilization to maturity. PlosOne 2013;8:e59270 doi: 10.1371/journal.pone.0059270 23555009 10.1371/journal.pone.0059270 PMC3598657

[33] Li L, Hur M, Lee J-Y, Zhou W, Song Z, Ransom N et al A systems biology approach toward understanding seed composition in soybean. BMC Genomics 2015;16:59 doi: 10.1186/1471-2164-16-S3-S9 25708381 10.1186/1471-2164-16-S3-S9 PMC4331812

[34] Ritchie SW, Hanway JJ, Thompson HE, Benson GO. How a Soybean Plant Develops In: Special Report n°53. Ames, Iowa: Iowa State University of Science and Technology: Cooperative Extension Service; 1985 p. 1–20.

[35] Rosnoblet C, Aubry C, Leprince O, Vu BL, Rogniaux H, Buitink J. The regulatory gamma subunit SNF4b of the sucrose non-fermenting-related kinase complex is involved in longevity and stachyose accumulation during maturation of *Medicago truncatula* seeds. Plant J. 2007;51:47–59. doi: 10.1111/j.1365-313X.2007.03116.x 17488238 10.1111/j.1365-313X.2007.03116.x

[36] Langmead B, Trapnell C, Pop M, Salzberg SL. Ultrafast and memory efficient alignment of short DNA sequences to the human genome. Genome Biology 2009;10:25–32. doi: 10.1186/gb-2009-10-3-r25 19261174 10.1186/gb-2009-10-3-r25 PMC2690996

[37] Love M, Huber W, Anders S. Moderated estimation of fold change and dispersion for RNA-seq data with DESeq2. Genome Biology 2014;15:550 doi: 10.1186/s13059-014-0550-8 25516281 10.1186/s13059-014-0550-8 PMC4302049

[38] Horvath S, Dong J. Geometric interpretation of gene coexpression network analysis PLoS Comput. Biol. 2008;4:e1000117 doi: 10.1371/journal.pcbi.1000117 18704157 10.1371/journal.pcbi.1000117 PMC2446438

[39] Ruijter JM, Ramakers C, Hoogaars WMH, Karlen Y, Bakker O, van den Hoff MJB et al Amplification efficiency: linking baseline and bias in the analysis of quantitative PCR data. Nucl. Acids Res. 2009;37:e45 doi: 10.1093/nar/gkp045 19237396 10.1093/nar/gkp045 PMC2665230

[40] Schmittgen TD, Livak KJ. Analyzing real-time PCR data by the comparative C(T) method. Nat. Protoc. 2008;3:1101–8. doi: 10.1038/nprot.2008.73 18546601 10.1038/nprot.2008.73

[41] Teixeira RN, Ligterink W, França-Neto JB, Hilhorst HWM, da Silva EAA. Gene expression profiling of the green seed problem in soybean. BMC Plant Biol. 2016;16:37 doi: 10.1186/s12870-016-0729-0 26829931 10.1186/s12870-016-0729-0 PMC4736698

[42] Lowell CA, Kuo TM. Oligosaccharide metabolism and accumulation in developing soybean seeds. Crop Sci. 1989; 29:459–65.

[43] Saravitz DM, Pharr DM, Carter TE Jr. Galactinol synthase activity and soluble sugars in developing seeds of four soybean genotypes. Plant Physiol. 1987;83:185–9. 16665199 10.1104/pp.83.1.185 PMC1056321

[44] Obendorf RL, Zimmerman AD, Zhang Q, Castillo A, Kosina SM, Bryant EG et al Accumulation of soluble carbohydrates during seed development and maturation of low-raffinose, low-stachyose soybean. Crop Sci. 2009;49:329–41. doi: 10.2135/cropsci2008.06.0370

[45] Sakuraba Y, Schelbert S, Park S-Y, Han S-H, Lee B-D, Besagni-Andrès C et al STAY-GREEN and chlorophyll catabolic enzymes interact at light-harvesting complex II for chlorophyll detoxification during leaf senescence in Arabidopsis. Plant Cel 2012;24:507–18. doi: 10.1105/tpc.111.089474 22366162 10.1105/tpc.111.089474 PMC3315229

[46] Cheng MC, Hsieh EJ, Chen JH, Chen HY, Lin TP. Arabidopsis RGLG2, functioning as a RING E3 ligase, interacts with AtERF53 and negatively regulates the plant drought stress response Plant Physiol. 2012;158:363–75. doi: 10.1104/pp.111.189738 22095047 10.1104/pp.111.189738 PMC3252077

[47] Angelovici R, Galili G, Fernie AR, Fait A. Seed desiccation: a bridge between maturation and germination. Trends Plant Sci. 2010;15:211–8. doi: 10.1016/j.tplants.2010.01.003 20138563 10.1016/j.tplants.2010.01.003

[48] Nakabayashi K, Okamoto M, Koshiba T, Kamiya Y, Nambara E (2005) Genome-wide profiling of stored mRNA in *Arabidopsis thaliana* seed germination: epigenetic and genetic regulation of transcription in seed. Plant J 41: 697–709. doi: 10.1111/j.1365-313X.2005.02337.x 15703057 10.1111/j.1365-313X.2005.02337.x

[49] Dietz K-J, Vogel MO, Viehhauser A. AP2/EREBP transcription factors are part of gene regulatory networks and integrate metabolic, hormonal and environmental signals in stress acclimation and retrograde signaling. Protoplasma 2010;245:3–14. doi: 10.1007/s00709-010-0142-8 20411284 10.1007/s00709-010-0142-8

[50] Huang Y, Feng C-Z, Ye Q, Wu W-H, Chen Y-F. *Arabidopsis WRKY6* transcription factor acts as a positive regulator of abscisic acid signaling during seed germination and early seedling development. PLoS Genet. 2016; 2:e1005833 doi: 10.1371/journal.pgen.1005833 26829043 10.1371/journal.pgen.1005833 PMC4734665

[51] Capella M, Ribone PA, Arce A L., Chan R L. *Arabidopsis thaliana* HomeoBox 1 (AtHB1), a Homedomain-Leucine Zipper I (HD-Zip I) transcription factor, is regulated by PHYTOCHROME-INTERACTING FACTOR 1 to promote hypocotyl elongation. New Phytol. 2015;207: 669–82. doi: 10.1111/nph.13401 25865500 10.1111/nph.13401

[52] Feurtado JA, Huang D, Wicki-Stordeur L, Hemstock LE, Potentier MS, Tsang EW et al The Arabidopsis C2H2 zinc finger INDETERMINATE DOMAIN1/ENHYDROUS promotes the transition to germination by regulating light and hormonal signaling during seed maturation. Plant Cell 2011;23:1772–94. doi: 10.1105/tpc.111.085134 21571950 10.1105/tpc.111.085134 PMC3123948

[53] Zhang Y, Du L, Xu R, Cui R, Hao J, Sun C et al Transcription factors SOD7/NGAL2 and DPA4/NGAL3 act redundantly to regulate seed size by directly repressing *KLU* expression in *Arabidopsis thaliana*. Plant Cell 2015;27:620–32. doi: 10.1105/tpc.114.135368 25783029 10.1105/tpc.114.135368 PMC4558667

[54] Duplan V, Rivas S. E3 ubiquitin-ligases and their target proteins during the regulation of plant innate immunity. Front. Plant Sci. 2014;13:45 doi: 10.3389/fpls.2014.00042 24592270 10.3389/fpls.2014.00042 PMC3923142

[55] Almoguera C, Prieto-Dapena P, Diaz-Martin J, Espinosa JM, Carranco R, Jordano J The HaDREB2 transcription factor enhances basal thermotolerance and longevity of seeds through functional interaction with HaHSFA9. BMC Plant Biol. 2009;9:75 doi: 10.1186/1471-2229-9-75 19545370 10.1186/1471-2229-9-75 PMC2706249

[56] Lisso J, Altmann T, Müssig C. The AtNFXL1 gene encodes a NF-X1 type zinc finger protein required for growth under salt stress. FEBS Lett. 2006;580:4851–6. doi: 10.1016/j.febslet.2006.07.079 16905136 10.1016/j.febslet.2006.07.079

[57] Schuster C, Gaillochet C, Lohmann JU. 2015. Arabidopsis *HECATE* genes function in phytohormone control during gynoecium development. Development 2015;142:3343–50. doi: 10.1242/dev.120444 26293302 10.1242/dev.120444 PMC4631749

[58] Kaufmann K, Muiño JM, Jauregui R, Airoldi CA, Smaczniak C, Krajewski P et al Target genes of the MADS transcription factor SEPALLATA3: integration of developmental and hormonal pathways in the *Arabidopsis* flower. PLoS Biol. 2009;7:e1000090 doi: 10.1371/journal.pbio.1000090 19385720 10.1371/journal.pbio.1000090 PMC2671559

[59] Yamaguchi N, Jeong CW, Nole-Wilson S, Krizek BA, Wagner D. AINTEGUMENTA and AINTEGUMENTA-LIKE6/PLETHORA3 Induce *LEAFY* Expression in response to auxin to promote the onset of flower formation in Arabidopsis. Plant Physiol. 2016;170:283–93. doi: 10.1104/pp.15.00969 26537561 10.1104/pp.15.00969 PMC4704571

[60] Fulcher N, Sablowski R. Hypersensitivity to DNA damage in plant stem cell niches. Proc. Natl. Acad. Sci. USA. 2009;106:20984–8. doi: 10.1073/pnas.0909218106 19933334 10.1073/pnas.0909218106 PMC2791609

[61] Waters ER. The evolution, function, structure and expression of the plant sHSPs. J. Exp. Bot. 2013;64:39–403. doi: 10.1093/jxb/ers355 23255280 10.1093/jxb/ers355

[62] Kaur H, Petla BP, Kamble NU, Singh A, Rao V, Salvi P et al Differentially expressed seed aging responsive heat shock protein OsHSP18.2 implicates in seed vigor, longevity and improves germination and seedling establishment under abiotic stress. Front. Plant Sci. 2015;6:713 doi: 10.3389/fpls.2015.00713 26442027 10.3389/fpls.2015.00713 PMC4568394

[63] Bilyeu KD, Wiebold WJ. Environmental stability of seed carbohydrate profiles in soybeans containing different alleles of the raffinose synthase 2 (RS2) gene. J. Agric. Food. Chem. 2016;64:1071–8. doi: 10.1021/acs.jafc.5b04779 26800264 10.1021/acs.jafc.5b04779

[64] de Souza Vigidal D, Willems L, van Arkel J, Dekkers BJ, Hilhorst HWM, Bentsink L. Galactinol as marker for seed longevity. Plant Sci. 2016;246:112–8. doi: 10.1016/j.plantsci.2016.02.015 26993241 10.1016/j.plantsci.2016.02.015

[65] Nishizawa A, Yabuta Y, Shigeoka S. Galactinol and raffinose constitute a novel function to protect plants from oxidative damage. Plant Physiol. 2008;147:1251–63. doi: 10.1104/pp.108.122465 18502973 10.1104/pp.108.122465 PMC2442551

[66] Padua GPD, França-Neto JDB, Carvalho MLMD, Costa O, Krzyzanowski FC, Costa NPD. Tolerance level of green seed in soybean seed lots after storage. Rev. Bras. Sementes 2007;29:128–38. doi: 10.1590/S0101-31222007000300015

[67] Ballesteros D, Walters C. Detailed characterization of mechanical properties and molecular mobility with dry seed glasses: relevance to the physiology of dry biological systems. Plant J. 2011;68:607–19. doi: 10.1111/j.1365-313X.2011.04711.x 21831210 10.1111/j.1365-313X.2011.04711.x

[68] Niedzielski M, Walters C, Luczak W, Hill LM, Wheeler LJ, Puchalski J. Assessment of variation in seed longevity within rye, wheat and the intergeneric hybrid triticale. Seed Sci. Res. 2009;19:213–24. doi: 10.1017/S0960258509990110

[69] Roberts EH, Ellis RH. Water and seed survival. Ann. Bot. 1989;63:39–52.

[70] Schwember AR, Bradford KJ. Quantitative trait loci associated with longevity of lettuce seeds under conventional and controlled deterioration storage conditions. J. Exp. Bot. 2010;61: 4423–36. doi: 10.1093/jxb/erq248 20693410 10.1093/jxb/erq248 PMC2955753

[71] Vertucci CW, Leopold AC. Bound water in soybean seed and its relation to respiration and imbibitional damage. Plant Physiol. 1984;75:114–7. 16663553 10.1104/pp.75.1.114 PMC1066845

