# Supplementary figures and images for: Molecular characterization of the acquisition of longevity during seed maturation in soybean
Source: PLoS One. 2017 Jul 12;12(7):e0180282. doi: 10.1371/journal.pone.0180282 (PMC5507495; doi:10.1371/journal.pone.0180282)

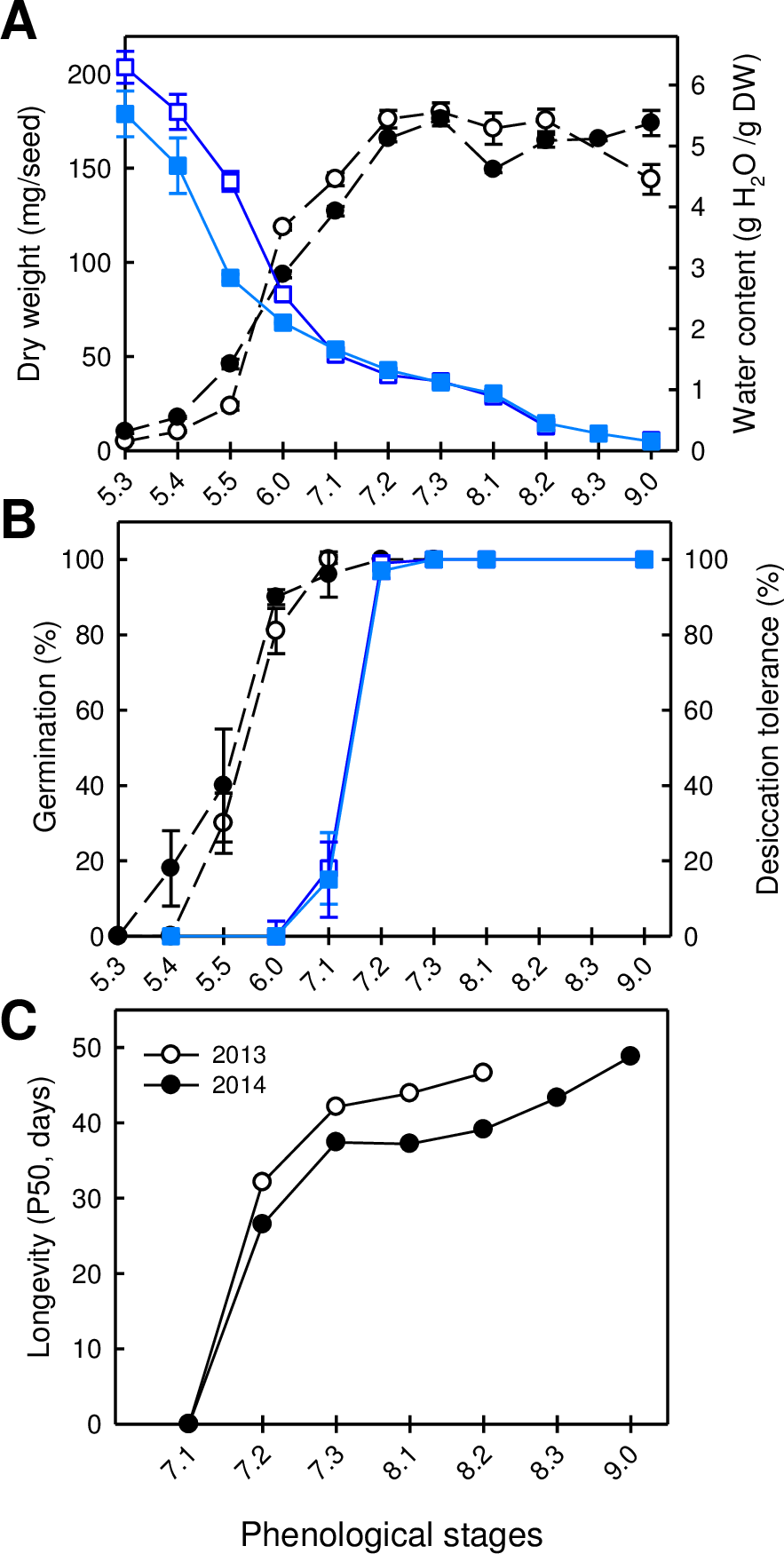

Supplement: S1 Fig — (A) Evolution of seed dry weight (black circles) and water content (blue squares). Data are the means (± SE) of 3 to 5 replicates of 20 seeds. (B) Acquisition of germination (black circles) and desiccation tolerance (blue squares), evaluated after fast drying to 10% moisture. (C) Acquisition of longevity as assessed by P50 (time necessary to obtain a loss of viability of 50% during storage 35°C and 75% RH). Data are the means (± SE) of 4 replicates of 25 seeds. Data are presented for 2013 (open symbols) and 2014 (closed symbols). (TIF) [file pone.0180282.s001.tif]
