# Supplementary material for: Molecular characterization of the acquisition of longevity during seed maturation in soybean
Source: PLoS One. 2017 Jul 12;12(7):e0180282. doi: 10.1371/journal.pone.0180282 (PMC5507495; doi:10.1371/journal.pone.0180282)
Supplement: S1 Table — (PDF) [file pone.0180282.s002.pdf]

S1. Table. Description of the reproductive phenological stages of soybean seeds according to Ritchie et al. 1982 and those added in this work.

Stages	Phenological stages according to Ritchie et al.		New phonological stages added in this work	
	Denomination	Description	Denomination	Description
$R_{5,1}$	Seed filling	Seeds ~ 5mm long in the pod (perceptible to touch)		
$R_{5,2}$	Seed filling	Seeds ~ 7mm long in the pod		
$R_{5.3}$	Seed filling	Seeds ~ 8mm long in the pod		
$R_{5,4}$	Seed filling	Seeds ~ 10mm long in the pod		
$R_{5,5}$	Seed filling	Seeds ~ 11mm long in the pod		
R_6	Full seed	Pod containing a green seed that fills the pod cavity at one of the four uppermost nodes on the main stem with a fully developed leaf		
R7	Begining maturity	one normal pod on the main stem that has reached its mature pod color		
$R_{7.1}$			Early mature green	Up to 50% of yellowing leaves and pods, green seeds
$R_{7.2}$			Physiological maturity	Between 51% and 75% yellow leaves and pods, green-yellowish seeds
$R_{7.3}$			Early mature yellow	More than 75% yellow leaves and pods, yellow seeds
R8	Full maturity	95% of the pods have reached their mature pod color		Not used
$R_{8.1}$			Mid mature yellow	Up to 50% defoliation, dark yellow pods, yellow seeds
$R_{8.2}$			Late mature yellow	50-80% defoliation, brown-yellow pods, yellow seeds
$R_{8.3}$			Late mature desiccating	More than 80% defoliation, brown pods and light brown seeds
R_9	Harvest maturity	No leaves, dry brown pods and light brown seeds		
