# Supplementary material for: Molecular characterization of the acquisition of longevity during seed maturation in soybean
Source: PLoS One. 2017 Jul 12;12(7):e0180282. doi: 10.1371/journal.pone.0180282 (PMC5507495; doi:10.1371/journal.pone.0180282)
Supplement: S2 Table — (DOCX) [file pone.0180282.s003.docx]

**S2 Table. List of primers combination used for gene expression validation of target genes by qRT-PCR**

| **Gene** | **Glyma2.0** | **Forward** | **Reverse** |
| --- | --- | --- | --- |
| 20S proteasome subunit* | Glyma.06g078500 | CACCAACACACGATACAACT | TCCCAACCACCAACAATTAACC |
| 60S Ribosomal protein L6* | Glyma.15g271300 | GGCAGAGAAGGAGGAGAA | ACCTAGCACCCAAGTAAGA |
| HSP 17.6a | Glyma.17g224900 | ACGAAAGAGGGACGAAGA | CATTCTCAGGCAGCACAA |
| HSP 17.6b | Glyma.14g100000 | TGCGGATGTGAAGGAATATC | AAGCACGTTGTCGTCTTC |
| HSP 21 | Glyma.08g318900 | AACATGCTGGTGGTGAAG | AGGGCTATCCTGTGGTTAT |
| HSFA3 | Glyma.03g191100 | CATCAGGTTGGTGGCAATA | GCATTAGCACACTCCTTTCT |
| HSFA6B | Glyma.03g157300 | GAGTGTCAGAGTTGGAAGTG | CCAGCCTCTCTTGTGATTG |

*Reference gene
